# Enhancing ZTMAF through federated trust learning, post quantum cryptography integration and seamless cross domain authentication handover

**DOI:** 10.3389/frai.2026.1910483

**Published:** 2026-09-25

**Authors:** Jeevan Yoganand, Riya Bansal V., Dishal L. S., Jeipratha P. N.

**Affiliations:** Department of Computer Science, School of Engineering, Vellore Institute of Technology (VIT), Chennai, Tamil Nadu, India

**Keywords:** authentication handover, cross-domain authentication, federated learning, intrusion detection, post-quantum cryptography (PQC), secure trust transmission, trust management, vehicular fog computing

## Abstract

Vehicular Fog Computing (VFC) has a major role to play in facilitating low-latency and real-time communication in Intelligent Transportation Systems (ITS). Nevertheless, there exist major issues in trust management, security, and cross-domain authentication, considering the dynamic nature of vehicular networks. The Zero-Trust Mobility-Aware Authentication Framework (ZTMAF) has been proposed to overcome issues in trust management, security, and cross-domain authentication. Nevertheless, there exist major issues in distributed intelligence, trust transmission, and handover, which have been addressed in this proposed framework. The proposed framework extends ZTMAF with three major advancements: Federated Trust Learning, PQC-inspired Secure Transmission, and Cross-Domain Authentication Handover. The proposed framework uses fog nodes to train a trust model in a distributed setting, allowing for scalability and privacy preservation. Additionally, Post-Quantum Cryptography inspired encryption would be utilized for secure trust transmission, as well as to remain compliant with future post-quantum encryption standards. A token-based handover mechanism will allow vehicles to make use of previously verified trust, thus lowering latency in cross-domain authentication. The proposed framework has been validated via multiple simulation scenarios, and its performance has been evaluated against different types of attacks (e.g., stealth, on/off, Sybil). The results of the evaluations show that trust stability has improved, that detection of behavior-based attacks has improved, and that the use of the token-based handover mechanism has reduced the authentication delay by 45%. The proposed framework represents a secure and scalable solution for next-generation vehicular networks, and it sheds light on the issues associated with detecting Sybil attacks as part of the process.

## 1 Introduction

With the integration of vehicular networks and Fog Computing, highly sophisticated Intelligent Transportation Systems (ITS) can now be developed with the need for real-time communication, low latency, and high reliability (Rose et al., 2020; Kaur et al., 2025). Vehicular Fog Computing (VFC) enables the offloading of computations and communication with the surrounding Fog Nodes by the vehicles, which can be used in various applications like avoiding collisions and traffic management and providing autonomous driving assistance(Ahmad, 2025); (Gong et al., 2023). However, the high mobility of the vehicles and the lack of connectivity and heterogeneous trust domains are the significant security challenges (Kang et al., 2018).

Traditional methods of authentication, like PKI and Blockchain-based systems, are facing challenges in the VFC environment due to the high computational overhead and the latency caused by the consensus mechanism of the Blockchain system. To overcome the challenges of traditional methods of authentication in the VFC environment, the proposed Zero-Trust Mobility Aware Authentication Framework (ZTMAF) uses a zero-trust model in which every node is continuously authenticated on the basis of contextual and behavioral characteristics.

Despite the advantages offered by ZTMAF, the framework has some limitations when used in vehicular networks. Firstly, the framework is limited to localized trust computation. This limits the framework's ability to use knowledge shared by more than one fog node. Secondly, the framework does not provide strong protection for the transmission of trust values. Thirdly, vehicles have to undergo repeated authentication when they enter a new fog domain. Fourthly, the evaluations performed on the framework are limited to basic attacks and do not completely show the effects of more complex attacks, such as stealth and on–off attacks.

To address the limitations of ZTMAF, this paper presents an improved framework based on federated learning, secure transmission of trust values, and reuse of authentication in multiple fog domains. The framework is based on Federated Trust Learning. With Federated Trust Learning, fog nodes will be able to collaboratively learn trust models using logistic regression. The nodes will use federated averaging to update the learned models.

For ensuring secure communication, a PQC-inspired encryption layer is added to the system, ensuring that trust values are securely transmitted. Although a full-fledged post-quantum computing approach is beyond the scope of this work, a simulation for secure transmission is included to analyze its viability and potential. Additionally, a Cross-Domain Authentication Handover is included, allowing vehicles to reuse trust tokens for different fog domains, resulting in improved system efficiency.

The major novelty of the proposed ZTMAF+ framework lies in the integration of four complementary implementation-level enhancements into the original Zero-Trust Mobility-Aware Authentication Framework. First, Federated Trust Learning enables collaborative trust model training across multiple fog nodes without exchanging raw vehicle data, thereby improving scalability and preserving privacy. Second, a PQC-inspired secure trust transmission layer protects the communication of trust information through simulated quantum-resistant mechanisms without claiming full post-quantum cryptographic implementation. Third, the proposed Cross-Domain Authentication Handover mechanism enables authenticated vehicles to seamlessly migrate between neighboring fog domains using reusable trust tokens, thereby reducing authentication latency and computational overhead. Finally, Temporal Trust Computation incorporates historical behavioral information into trust estimation, improving robustness against dynamic and sophisticated attacks. Collectively, these enhancements distinguish the proposed ZTMAF+ framework from the original ZTMAF by providing improved scalability, secure trust exchange, efficient authentication continuity, and stronger resilience against advanced vehicular attacks.

We validate the proposed system's performance using simulation results, which include a detailed analysis of vehicle behavior, trust evolution and many different attacks. The system simulations have been tested on the attack types of stealth, on–off, and Sybil. The results indicate that while the proposed system performs well to defend against behavioral attacks, identity-based attacks remain an issue and future research will be needed to improve performance in this area.

Unlike the original Zero-Trust Mobility-Aware Authentication Framework (ZTMAF), which primarily focuses on context-aware trust evaluation and mobility-aware authentication within individual fog domains, the proposed ZTMAF+ extends the framework through four implementation-level enhancements. First, Federated Trust Learning enables collaborative trust model 2 training across multiple fog nodes using federated averaging while preserving local data privacy. Second, a PQC-inspired secure trust transmission layer is incorporated to simulate the secure exchange of trust values and evaluate the feasibility of integrating post-quantum cryptographic workflows into vehicular fog environments. Third, a token-based cross-domain authentication handover mechanism enables authenticated vehicles to reuse previously verified trust information when transitioning between fog domains, thereby reducing authentication latency. Finally, the framework introduces temporal trust computation and evaluates its robustness against stealth, on–off, and Sybil attacks using a large-scale simulated vehicular dataset. These enhancements collectively improve the scalability, mobility support, and security evaluation capabilities of the original ZTMAF while maintaining compatibility with its zero-trust authentication model.

## 2 Related work

### 2.1 Zero-trust architectures for vehicular networks

Rose et al., as well as NIST SP 800-207, describe the zero-trust security model by using the “never trust, always verify” framework to remove implicit trust based on some level of verification (Rose et al., 2020; Gong et al., 2023). Kaur et al. (2025) demonstrated how the zero-trust security model could be adapted to fog-based healthcare systems by using a zero-trust framework in conjunction with blockchain technology to demonstrate that such models can be used effectively with distributed systems. The Zero-Trust Mobility-Aware Authentication Framework (ZTMAF) was developed by Ahmad, incorporating context-based authentication and lightweight cryptographic protocols for vehicular fog computing (Ahmad, 2025). Gong et al. (2023) presented a detailed survey on edge intelligence for intelligent transportation systems, providing a foundational understanding for architectural design. Although the ZTMAF is a significant advancement, providing a means to include mobility into authentication, it is still a local trust evaluation system without the means for cross-system knowledge sharing or quantum-resistant cryptography (Kang et al., 2018; Gao et al., 2020).

### 2.2 Trust and reputation management in fog-based vehicular systems

Dynamic trust evaluation is a significant issue for secure vehicular communication, and Loffi et al. presented a number of mutual authentication protocols for IoT applications in a fog computing environment, depending on the behavior pattern of the device (Loffi et al., 2019). Yang et al. (2018) presented a new approach to blockchain-based decentralized trust management for vehicular networks, where the immutability of the system is shown. Another approach to a blockchain-based reputation system for vehicular edge computing is presented by Kang et al. (2018), though the consensus process limits its application to real-time systems. Lim et al. (2020) presented a number of applications for federated learning in mobile edge networks, establishing a number of frameworks for distributed intelligence. Alnaim and Alwakeel (2025) presented a number of zero-trust mechanisms for distributed edge and fog computing security in 6G networks, focusing on the security requirements for 6G networks. Ashfaq et al. present the challenges and solutions with regard to the improvement of zero-trust security in edge computing. This establishes the significance of behavior-aware evaluation but remains isolated within a single fog domain without privacy-preserving trust aggregation (Ashfaq et al., 2024; Zhang Y. et al., 2021; Yadav and Peroumal, 2023; Chaba et al., 2017).

### 2.3 DHT federated learning for distributed trust aggregation

Federated learning facilitates distributed model training in edge networks, ensuring privacy preservation, as shown in Yang et al. (2018)'s work on blockchain-based decentralized trust management in vehicular networks, which utilized distributed consensus mechanisms. The work carried out by Lim et al. (2020) provides a theoretical basis for federated learning in mobile edge networks, covering FedAvg convergence behavior and communication efficiency. The work carried out by Alnaim and Alwakeel (2025) provides a basis for understanding zero trust mechanisms in securing distributed edge and fog computing networks in 6G networks, providing a context for federated learning integration in security architectures. The work carried out by Ashfaq et al. (2024) provides an analysis of zero trust security in edge computing networks, highlighting integration opportunities with federated learning architectures. Zhang Y. et al. (2021) proposed an efficient privacy-preserving authentication scheme for V2G networks, highlighting cryptographic techniques that could be utilized in distributed trust aggregation. Although there has been significant progress in federated learning, existing applications only support generalized prediction, with security-specific trust modeling unexplored, including zero trust authentication integration, as shown in existing applications, including (Yadav and Peroumal, 2023; Chaba et al., 2017).

### 2.4 Authentication protocols for vehicular fog computing

Yadav and Peroumal (2023) presented an efficient and lightweight mutual authentication and key exchange protocol for roaming vehicles, which is computationally efficient and suitable for embedded systems. Chaba et al. (2017) presented a secure and efficient key delivery in VANETs with the aid of cloud and fog computing, which operates with low latency. Alkim et al. (2016) presented the lattice-based key exchange with the New Hope protocol and established the practical feasibility of quantum-resistant cryptography. CRYSTALS-Kyber and CRYSTALS-Dilithium are NIST-standard post-quantum cryptographic schemes suitable for inclusion in security protocols (Bos et al., 2018; Ducas et al., 2018). Moura et al. (2025) presented the application of post-quantum cryptography in the context of IoT systems and the corresponding performance considerations. Zerraza et al. (2024) presented an efficient and lightweight authentication and access control scheme suitable for the IoT paradigm. All the above schemes are based on the use of quantum-vulnerable schemes and the assumption of network participation, which is not suitable for highly mobile networks.

### 2.5 Post-quantum cryptography for edge and vehicular environments

Alkim et al. presented the practical viability of post-quantum cryptography through the introduction of the lattice-based key exchange scheme, New Hope, by Alkim et al. Bos et al. formalized the module lattices for a CCA-secure key encapsulation scheme, known as CRYSTALS-Kyber, which is a NIST-standardized scheme. Ducas et al. presented a practical scheme for digital signatures, known as CRYSTALS-Dilithium, to form a complete post-quantum cryptographic scheme. Moura et al. presented a detailed analysis on the application of PQC for IoT and edge computing, highlighting performance trade-offs for these applications. Roy et al. presented a new hybrid authentication scheme for IoT applications, utilizing lattice-based cryptography for edge computing and fog networks. Moon et al. presented an analysis on edge-fog enhanced post-quantum network security, including applications, challenges, and solutions for next-generation network security. It is evident that the performance evaluation for these applications does not take into consideration vehicular movement patterns, real-time authentication requirements, and workload distribution for the application of fog computing, and hence the application of zero-trust authentication remains an unexplored area, especially for vehicular networks, as the lifespan of connected vehicles is 10–15 years.

### 2.6 Handover and mobility management in secure vehicular communications

In traditional solutions, full re-authentication is necessary when entering new fog domains, leading to latency spikes that are problematic for continuous applications. Bahreini et al. proposed VECMAN, an energy-aware resource management framework for vehicular edge computing networks, considering mobility factors. Hou et al. (2016) defined the basic viewpoint of vehicles as infrastructure in vehicular fog computing, outlining the principles of architecture for mobility management. Huang et al. (2017) investigated the architecture, scenarios, and security issues of vehicular fog computing, with handover being one of the important issues. Ning et al. (2019) researched vehicular fog computing for facilitating real-time traffic management, with mobility management being an important factor. (Nazih et al., 2024) performed a review of security and trustworthiness challenges in vehicular fog computing, conducting an extensive analysis of security related to mobility management. Liu et al. (2024) developed a multidimensional trust evaluation model that increases the security of a network in fog computing. Alemneh et al. (2020) created a two-way trust management mechanism that enables two-way establishment of trusts in a fog computing environment. Ogundoyin and Kamil (2021) introduced a trust management system for fog computing service, supporting an ongoing authentication methodology. Zhang G. et al. (2021) introduced a hidden data attack detection technique utilizing fog computing with trust assessment methods to solve advanced attack methods for secure cloud and fog-enabled communications. Roy et al. (2024) introduced a new hybrid authentication protocol using lattice-based cryptography for IoT devices in a fog computing environment, demonstrating a new approach to handover in a post-quantum computing environment. (Moon et al., 2025) provided a comprehensive study on edge-fog enhanced post-quantum security in a network environment, addressing various aspects such as applications, challenges, and solutions for mobility management in a wireless environment. The ZTMAF framework recognizes the problem associated with mobility management, relying on local trust evaluation and requiring trust rebuilding during a transition from one domain to another (Ahmad, 2025).

### 2.7 Comparison with the original ZTMAF framework

Although the original Zero-Trust Mobility-Aware Authentication Framework (ZTMAF) provides a robust trust-based authentication mechanism for vehicular fog computing, it exhibits several limitations in terms of scalability, cross-domain authentication continuity, and adaptive trust learning. The proposed ZTMAF+ extends the original framework by integrating Federated Trust Learning, a PQC-inspired secure trust transmission mechanism, Cross-Domain Authentication Handover, and Temporal Trust Computation. These enhancements significantly improve privacy preservation, authentication efficiency, trust robustness, and scalability while maintaining compatibility with the original zero-trust architecture.

Table 1 summarizes the major architectural and functional differences between the original ZTMAF and the proposed ZTMAF+ framework.

**Table 1:** Comparison between the original ZTMAF and proposed ZTMAF+ framework.

Feature	Original ZTMAF	Proposed ZTMAF+
Trust computation	Instantaneous trust evaluation	Temporal trust computation using historical behavior
Learning strategy	Centralized trust evaluation	Federated trust learning across fog nodes
Trust transmission	Traditional encrypted communication	PQC-inspired secure trust transmission layer
Authentication handover	Fresh authentication required after domain change	Cross-domain authentication token reuse
Privacy preservation	Limited	Enhanced through federated learning
Scalability	Suitable for moderate deployments	Improved for large-scale vehicular fog environments
Attack handling	Basic trust attacks	Supports stealth, on–off and Sybil attack evaluation
Overall framework	Zero-trust authentication	Enhanced zero-trust authentication with learning and secure trust exchange

### 2.8 State-of-the-art comparison of existing trust management frameworks

Several trust management and authentication frameworks have been proposed for Vehicular Ad Hoc Networks (VANETs) and Vehicular Fog Computing (VFC). Existing approaches primarily focus on trust evaluation, authentication, or privacy preservation; however, only a few integrate multiple security mechanisms within a unified zero-trust architecture. The proposed ZTMAF+ framework distinguishes itself by combining Federated Trust Learning, PQC-inspired secure trust transmission, Cross-Domain Authentication Handover, and Temporal Trust Computation within a single scalable framework. Furthermore, the framework is evaluated against advanced vehicular attacks including stealth, on–off, and Sybil attacks, providing enhanced security and authentication robustness (Table 2).

**Table 2:** State-of-the-art comparison of existing trust management frameworks.

Framework	Federated learning	PQC support	Cross-domain handover	Advanced attack evaluation	Zero-trust authentication
Traditional PKI-based authentication	X	X	X	X	X
Blockchain-based trust models	X	X	Partial	Partial	X
Machine learning-based trust frameworks	X	X	X	✓	X
Original ZTMAF	X	X	X	Partial	✓
Proposed ZTMAF+	✓	✓(Inspired)	✓	✓	✓

## 3 System model

### 3.1 Network architecture

The vehicular fog computing environment is modeled as a dynamic graph:


$$\begin{array}{c} G=\left(N,L\right),\;N=U\cup F \end{array}$$ (1)

where *N* is the set of nodes, *U* = {*u*_1_, *u*_2_, …, *u*_*M*_} is the set of vehicles, *F* = {*f*_1_, *f*_2_, …, *f*_*K*_} is the set of fog nodes, and *L* represents authenticated communication links.

Each vehicle *u*_*i*_ interacts with nearby fog nodes *f*_*j*_ for authentication and trust evaluation. The system operates in a multi-domain environment, where each fog node represents a distinct trust domain.

#### 3.1.1 System workflow

The end-to-end process is defined as:

*u*_*i*_ → Trust Computation → Secure Transmission → *f*_*j*_ → Federated Learning → Decision → Token Generation → Handover

### 3.2 Trust model

Each vehicle maintains a dynamic trust score based on multiple behavioral and contextual parameters.

#### 3.2.1 Context vector


$$\begin{array}{c} c_{i}\left(t\right)=\left[s_{i}\left(t\right),a_{i}\left(t\right),r_{i}\left(t\right),p_{i}\left(t\right)\right] \end{array}$$ (2)

where *s*_*i*_(*t*) denotes the speed of vehicle *i* at time *t*, *a*_*i*_(*t*) represents its acceleration, *r*_*i*_(*t*) indicates the degree of rule compliance, and *p*_*i*_(*t*) corresponds to the packet delivery ratio reflecting communication reliability.

#### 3.2.2 Trust computation


$$\begin{array}{c} T_{i}^{raw}\left(t\right)=\underset{k}{\sum }w_{k}f_{k}\left(c_{i}\left(t\right)\right) \end{array}$$ (3)

where *w*_*k*_ denotes the weights assigned to each parameter and *f*_*k*_(·) represents normalization and nonlinear scaling functions.

#### 3.2.3 Temporal smoothing


$$\begin{array}{c} T_{i}\left(t+1\right)=\alpha T_{i}\left(t\right)+\left(1-\alpha \right)T_{i}^{raw}\left(t\right) \end{array}$$ (4)

where α ∈ (0, 1) controls the influence of historical trust.

### 3.3 Federated learning model

To enable collaborative trust learning, fog nodes train local models and share parameters using federated learning.

#### 3.3.1 Local model

Each fog node trains a logistic regression classifier:


$$\begin{array}{c} P\left(y_{i}=1|x_{i}\right)=\sigma \left(w^{T}x_{i}+b\right) \end{array}$$ (5)

where *x*_*i*_ is the feature vector, *y*_*i*_ is the binary label (benign/malicious), and σ(·) denotes the sigmoid activation function.

#### 3.3.2 Federated aggregation


$$\begin{array}{c} w_{global}=\overset{K}{\underset{j=1}{\sum }}\frac{n_{j}}{N}w_{j} \end{array}$$ (6)

$$\begin{array}{c} N=\overset{K}{\underset{j=1}{\sum }}n_{j} \end{array}$$ (7)

where *w*_*j*_ denotes the local model weights and *n*_*j*_ is the number of samples at node *j*.

#### 3.3.3 Decision function

The final classification decision is:


$$\begin{array}{c} D_{i}=\{\begin{array}{cc} \text{ACCEPT}, & P\left(y_{i}=1\right)<\tau \\ \text{REJECT}, & P\left(y_{i}=1\right)\geq \tau \end{array} \end{array}$$ (8)

### 3.4 PQC-inspired secure transmission layer

To protect trust values during communication, a PQC-inspired secure transmission layer is introduced.

#### 3.4.1 Encryption and decryption


$$\begin{array}{c} T_{i}^{enc}=E\left(T_{i}\right) \end{array}$$ (9)

$$\begin{array}{c} T_{i}^{dec}=D\left(T_{i}^{enc}\right) \end{array}$$ (10)

where *E*(·) and *D*(·) are encryption and decryption functions, respectively.

#### 3.4.2 Design characteristics

The proposed design models the secure transmission of trust values through a PQC-inspired simulation layer. The system is architected to represent the potential integration of quantum-resistant cryptographic techniques such as lattice-based cryptography in future implementations. An important requirement of the proposed system is the integrity of the managed data during the entire process of trust management, which can be represented by the condition: $T_{i}^{dec}=T_{i}$. This equation ensures lossless reconstruction, and it is important to note that the trust value *T*_*i*_ originally assigned by an entity is reconstructed at the receiver after the simulated encryption and decryption process without alteration of the trust metric.

#### 3.4.3 Implementation considerations

The PQC layer is implemented using simulated mechanisms rather than an actual post-quantum cryptographic mechanism. Instead, an abstraction of simulated functions represents the sequence of encryption, transmission, and decryption activities that would occur in a cryptographic layer. This allows the performance of the secure transmission workflow to be evaluated without the computational expense associated with implementing an actual post-quantum cryptographic mechanism. No standardized post-quantum cryptographic algorithms, such as CRYSTALS-Kyber or CRYSTALS-Dilithium, are implemented in this work, and therefore no claims are made regarding quantum-resistant security guarantees. The proposed layer should instead be interpreted as a simulation-level architectural abstraction for evaluating the integration of future post-quantum secure transmission within the ZTMAF+ framework.

To clarify the implementation scope, the proposed framework does not implement standardized post-quantum cryptographic algorithms such as CRYSTALS-Kyber, CRYSTALS-Dilithium, or other NIST-approved PQC schemes. Instead, the framework introduces a PQC-inspired secure trust transmission layer that emulates the architectural principles of quantum-resistant communication while maintaining compatibility with conventional cryptographic mechanisms. The primary objective of this layer is to demonstrate the feasibility of integrating future post-quantum security concepts into zero-trust vehicular fog computing environments rather than claiming complete quantum-resistant security. Consequently, the proposed framework should be interpreted as an implementation-oriented proof of concept that provides a migration path toward future standardized PQC deployment.

### 3.5 Cross-domain authentication handover

A token-based authentication reuse mechanism is implemented to support mobility across multiple fog domains.

#### 3.5.1 Handover detection


$$\begin{array}{c} f_{prev}\left(u_{i}\right)\neq f_{curr}\left(u_{i}\right) \end{array}$$ (11)

#### 3.5.2 Token generation


$$\begin{array}{c} Token_{i}=H\left(ID_{i}∥T_{i}∥t\right) \end{array}$$ (12)

where *H*(·) denotes a hash function and *t* denotes a timestamp.

#### 3.5.3 Token-based authentication

If the car enters a different fog environment, then it can reuse the previous trust value and avoid having to go through the complete authentication process since it has a valid token. Conversely, if no valid token is supplied, then the normal authentication process will still occur to verify that the vehicle is legitimate.

#### 3.5.4 Latency optimization


$$\begin{array}{c} \lambda_{handover}<\lambda_{reauth} \end{array}$$ (13)

By reducing authentication delay, computational overhead, and repeated trust evaluations, the proposed mechanism improves overall authentication efficiency in highly dynamic vehicular environments.

## 4 Proposed methodology

This section describes the full functional operations of the proposed model, incorporating through a single end-to-end operation, trust computing, federated learning, secure transmission and cross-domain authentication.

### 4.1 End-to-end workflow

The stages of the suggested architectural model are illustrated as follows and shown in Figure 1:

**Data capture:** the vehicles have to continually capture the parameters in order for them to collect information such as Speed, Acceleration, Rule Compliance, and Successful Communication.

**Local trust score calculation:** the weighted behavioral features are the input parameters using which the first local trust score is being calculated.

**Secure Trust Score Transmission:** the local trust score is then processed by the PQC-inspired secure transmission layer, represented as an encrypted trust value, and forwarded to the relevant fog node as part of the simulated secure-transmission workflow.

**Fog node processing:** the local trust score which is sent by the vehicle is decrypted by the fog node. The vehicle's predicted trust score is obtained based on the data it holds in the local trust model.

**Federated learning update:** the fog node will continue to update the global model parameters using the vehicle in the federated learning model.

**Trust prediction and classification:** the global model will be able to predict the trust score of the vehicle, which will estimate the chances of the vehicle committing a malicious act (prediction).

**Vehicle authentication decision:** the predicted trust score obtained using the global model will be used in the authentication process of the vehicle.

**Token generation:** if the vehicle is accepted, the system generates the trust token, ready to be reused.

**Cross-domain handover:** the token is sent to the new fog domain, bypassing the authentication process, thus reducing latency.

**Figure 1:**
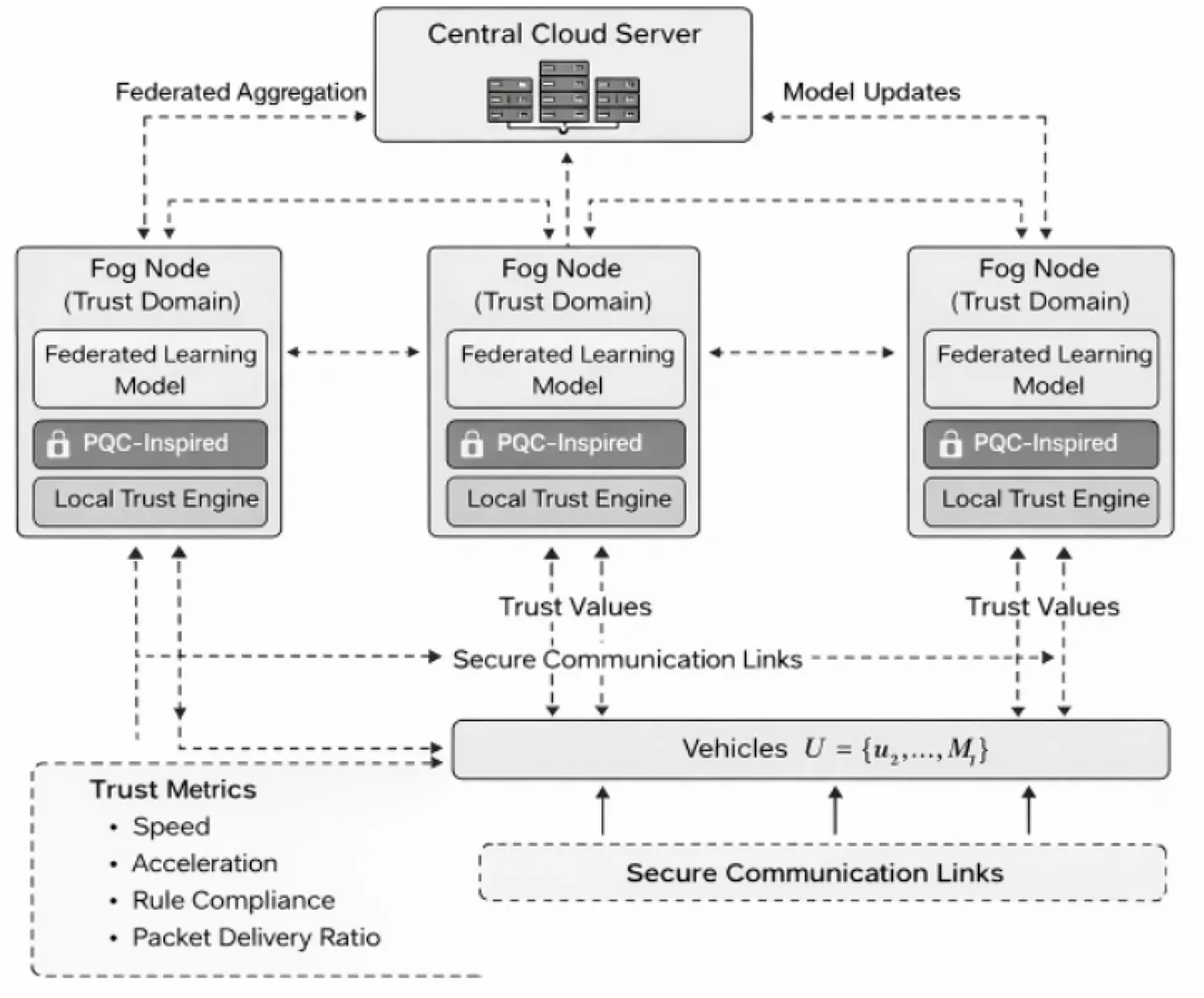
ZTMAF+ architecture diagram.

### 4.2 Trust computation model

The trust score is computed as a weighted combination of behavioral parameters:


$$\begin{array}{c} T_{i}^{raw}\left(t\right)=w_{1}s_{i}\left(t\right)+w_{2}a_{i}\left(t\right)+w_{3}r_{i}\left(t\right)+w_{4}p_{i}\left(t\right) \end{array}$$ (14)

where *s*_*i*_(*t*), *a*_*i*_(*t*), *r*_*i*_(*t*), and *p*_*i*_(*t*) denote speed, acceleration, rule compliance, and packet delivery ratio, respectively.

#### 4.2.1 Temporal update

To ensure stability and robustness:


$$\begin{array}{c} T_{i}\left(t+1\right)=\alpha T_{i}\left(t\right)+\left(1-\alpha \right)T_{i}^{raw}\left(t\right) \end{array}$$ (15)

This reduces sensitivity to short-term fluctuations and mitigates intermittent attack patterns.

### 4.3 Federated trust aggregation

Each fog node trains a local logistic regression model using its local dataset.

#### 4.3.1 Local training


$$\begin{array}{c} w_{j}\leftarrow \arg\min L\left(w_{j}\right) \end{array}$$ (16)

where *L*(·) represents the classification loss function.

#### 4.3.2 Global aggregation


$$\begin{array}{c} w_{global}=\overset{K}{\underset{j=1}{\sum }}\frac{n_{j}}{N}w_{j} \end{array}$$ (17)

This approach facilitates collaborative learning among the fog nodes, which in turn improves the generalization of the model while maintaining data privacy through the avoidance of direct data sharing.

#### 4.3.3 Prediction score


$$\begin{array}{c} {ŷ}_{i}=\sigma \left(w_{global}x_{i}\right) \end{array}$$ (18)

The output ŷ_*i*_ is stored as the prediction score and used for authentication decisions.

### 4.4 Secure trust transmission

The PQC-inspired layer ensures secure communication of trust values.


$$\begin{array}{c} T_{i}^{enc}=E\left(T_{i}\right) \end{array}$$ (19)

$$\begin{array}{c} T_{i}^{dec}=D\left(T_{i}^{enc}\right) \end{array}$$ (20)

The important characteristics of the proposed framework are the provision of confidentiality to the exchanged trust values, which are protected from unauthorized interception during the exchange process. Moreover, the system provides integrity to the exchanged values by reconstructing them without any loss during the process, which can be represented as :


$$\begin{array}{c} T_{i}^{dec}=T_{i} \end{array}$$ (21)

Finally, the system simulates the provision of compatibility with quantum-resistant cryptographic systems, such as lattice-based systems, which protects the system from potential quantum attacks in the future.

### 4.5 Authentication decision logic

The classification decision is based on a predefined threshold:


$$\begin{array}{c} D_{i}=\{\begin{array}{cc} \text{ACCEPT}, & P\left(y_{i}=1\right)<\tau \\ \text{REJECT}, & P\left(y_{i}=1\right)\geq \tau \end{array} \end{array}$$ (22)

Here, *ACCEPT* denotes a benign vehicle, while *REJECT* denotes a malicious vehicle.

### 4.6 Cross-domain handover mechanism

The framework supports efficient authentication across fog domains using token-based trust reuse.

#### 4.6.1 Handover detection


$$\begin{array}{l} handover=\{\begin{array}{ll} 1, & f_{prev}\left(u_{i}\right)\neq f_{curr}\left(u_{i}\right)\\ 0, & \text{otherwise} \end{array} \end{array}$$ (23)

#### 4.6.2 Token-based authentication

A valid token will allow a vehicle to use its previously calculated trust value in a new domain. Therefore, it will not be necessary to repeat the authentication process for that vehicle once it has entered that new domain. If there is no valid token, the vehicle will need to go through the normal authentication process to verify it is a real vehicle.

#### 4.6.3 Latency optimization


$$\begin{array}{c} \lambda_{handover}<\lambda_{reauth} \end{array}$$ (24)

As a result of these changes to the system, the authentication delay has been reduced, the scalability of the system has increased, and the computational cost of authenticating users has been decreased, improving the efficiency of the system overall.

### 4.7 Implementation details

The proposed ZTMAF+ framework was implemented using an integrated simulation environment consisting of SUMO, NS-3, Python, and TraCI. SUMO was employed to generate realistic vehicular mobility traces, while NS-3 simulated the vehicular communication network, including vehicle-to-vehicle (V2V) and vehicle-to-infrastructure (V2I) interactions. TraCI served as the communication interface between SUMO and NS-3, enabling real-time synchronization of vehicle positions, mobility events, and network behavior.

During each simulation interval, vehicle mobility information obtained from SUMO was transferred to NS-3 through TraCI, where trust computation and authentication decisions were performed. Each fog node periodically collected trust-related attributes, including communication behavior, packet forwarding statistics, authentication history, and mobility consistency. These attributes were processed by the Temporal Trust Computation module to calculate updated trust scores.

The Federated Trust Learning component was implemented using distributed local model training at individual fog nodes. Rather than exchanging raw vehicle data, only model parameters were periodically aggregated to construct a global trust prediction model while preserving data privacy. This federated learning process significantly reduced communication overhead and enabled scalable trust management across multiple fog domains.

The authentication workflow consists of vehicle registration, trust evaluation, authentication request verification, trust token generation, secure trust transmission, and cross-domain authentication handover. Vehicles with trust scores exceeding the predefined threshold are granted authentication tokens that can be securely reused during handover between neighboring fog domains, thereby minimizing repeated authentication latency.

The implementation was developed using Python for trust computation and machine learning, NS-3 for network simulation, SUMO for traffic simulation, TraCI for simulator synchronization, Scikit-learn for Logistic Regression and federated model training, NumPy and Pandas for data processing, and Matplotlib for visualization of experimental results.

## 5 Attack model

This section describes the adversarial scenarios considered in the proposed framework. To comprehensively evaluate system robustness, three representative attack models are implemented: Stealth Attacks, on–off Attacks, and Sybil Attacks.

### 5.1 Stealth attack

A stealth attack is characterized by gradual and subtle malicious behavior intended to evade detection mechanisms by maintaining trust values close to those of legitimate vehicles.

#### 5.1.1 Attack behavior

The attacker introduces minor modifications to behavioral parameters while maintaining an acceptable level of compliance with system rules, thereby avoiding abrupt behavioral changes that may trigger detection mechanisms.

#### 5.1.2 Mathematical representation


$$\begin{array}{c} c_{i}^{attack}\left(t\right)=c_{i}^{normal}\left(t\right)-\varepsilon \end{array}$$ (25)

where ε is a small perturbation factor.

#### 5.1.3 Impact on trust


$$\begin{array}{c} T_{i}\left(t\right)\approx T_{normal}\left(t\right)-\delta \end{array}$$ (26)

where δ is a small value that allows the attacker to remain within acceptable trust thresholds.

#### 5.1.4 Challenges

The difficulty in detecting this is because there is little deviation from the normal behavior, and there is overlap in the trust distribution, resulting in a high number of false negatives and hence a low level of recall.

### 5.2 ON–off attack

The on–off attack alternates between benign and malicious behavior to exploit temporal trust mechanisms.

#### 5.2.1 Attack behavior

The attack alternates between periods of normal behavior for trust gain and malicious behavior for executing the attack.

#### 5.2.2 Mathematical representation


$$\begin{array}{c} c_{i}\left(t\right)=\{\begin{array}{cc} c_{benign}, & t\in T_{on}\\ c_{malicious}, & t\in T_{off} \end{array} \end{array}$$ (27)

#### 5.2.3 Impact on trust


$$\begin{array}{c} T_{i}\left(t+1\right)=\alpha T_{i}\left(t\right)+\left(1-\alpha \right)\psi \left(c_{i}\left(t\right)\right) \end{array}$$ (28)

Due to temporal soothing: trust degradation is delayed and attacker retains moderate trust.

#### 5.2.4 Challenges

This attack takes advantage of temporal averaging and the delay in the adaptation of trust, making the malicious behavior less conspicuous over short periods. The solution to this problem is the recognition of patterns over time and global learning mechanisms to effectively capture the patterns in the behavior.

### 5.3 Sybil attack

A party that wants to harm a system can perform something called a Sybil attack, where they create numerous (but fake) identities to gain control over the system as if they were actually multiple participants in that system. In this case, one malicious party creates many (virtual) identities representing “vehicles” and can make these identities act like participants in a network by complying with some aspects of the system to establish a stake in it over time and to build trust as more participants use their services. With multiple identities and trust-building activities, the malicious party gains the ability to manipulate the process of evaluating trust through their coordinated actions, thereby allowing them to influence trust levels without having to act maliciously individually. The distributed nature of the attack makes it very difficult for the malicious actions to be detected because the coordinated behavior will appear “legitimately” under most situations; this is especially true in behavior-based trust models.

#### 5.3.1 Mathematical representation


$$\begin{array}{c} U_{sybil}=\left\{u_{1},u_{2},\ldots ,u_{k}\right\} \end{array}$$ (29)

where all identities relate to a single malicious entity.

#### 5.3.2 Impact on trust


$$\begin{array}{c} T_{group}=\frac{1}{k}\overset{k}{\underset{i=1}{\sum }}T_{i} \end{array}$$ (30)

As a result, this permits dispersal of trust build up about multiple identities, thereby allowing malicious actors can successfully hide what their real intentions are by creating an appearance of legitimacy through dispersal type behaviors. The absence of distinctive behavior patterns makes it extremely difficult to identify malicious activities based solely on the actions exhibited, since this attack relies on taking advantage of vulnerabilities related to their respective identities, not showing distinct behavioral patterns. Trust can be wrongfully increased when several of these types of threat actors are collaborating with one another, which ultimately results in serious increases in false negatives. Therefore, this illustrates that behavioral based detection methods may be insufficient for detecting threats related to identity. Thusly, this leads to demonstrating the necessity for utilizing identity based detection methodologies to assist in preventing attacks against legitimate entities.

## 6 Experimental setup

This section will detail the simulation environment, the dataset characteristics used, and the evaluation criteria used to assess the performance of the proposed framework.

### 6.1 Simulation environment

The evaluation of the proposed system is performed in a custom simulation framework consisting of a simulation framework that executes mobile vehicle simulation, trust calculation, federated learning, and authentication methods.

#### 6.1.1 Simulation configuration

- Simulation type: discrete-time vehicular simulation.
- Total samples: ~1.29 million records.
- Entities:
  − Vehicles (dynamic nodes).
  − Fog nodes (distributed processing units).
- Architecture: multi-domain vehicular fog environment.
- Attack types: stealth, on–off, Sybil.

#### 6.1.2 System components

**Trust engine:** computes multi-parameter trust with time-oriented smoothing.

**Federated learning module:** log linear models are trained locally at fog nodes then jointly aggregated nationally.

**Security layer:** PQC-inspired encryption of trust values.

**Authentication module:** uses decision making based on thresholds of prediction score.

**Handover mechanism:** token-based authentication for reuse of authenticated tokens among the multiple fog domains.

#### 6.1.3 Simulation workflow

Creating a simulated process begins by generating context based on what data is needed to calculate a vehicle's trusted value; after determining this valued assessment, it securely transmits them to fog nodes containing relevant contextual information about the location of the vehicle to whom it sends it, that subsequently provide information on how a vehicle trusts its identity by using federated learning to update the model's global parameters. If that vehicle's trusted value has been previously identified via its context as being valid, it is granted access to the network or will not have access granted. Tokens are also used during authentication to grant vehicles access through different locations which further optimizes both the authentication process as well as decreases the amount of computation required to manage a network access authentication process.

### 6.2 Dataset description

The evaluation is based on a comprehensive simulation dataset stored in CSV format. Sample rows from the dataset are presented in Tables 3–6 below.

**Table 3:** Input behavioral parameters for trust computation.

Step	vehicle_id	Speed	Acceleration	Rule_compliance	pdr
0	1	4.588526752	2.558902368	0.176035116	0.9
10	46	11.5937964	1.396269967	0.738046684	0.828986683
100	924	10.52863386	1.622069961	0.780961259	0.9
200	1144	2.449728834	–0.108413349	0.202463973	0.974867394
300	879	0.004220618	–0.003293081	0.00028742	0.9
400	2818	0.249964068	–0.822931683	0.010190245	0.9
500	852	0.104155045	–0.035991178	0.007839585	0.996918366
600	623	0.013097966	–0.006871035	0.000836344	0.9
700	1679	0.247760825	–0.05487944	0.015611172	0.968305844
800	1522	0.000171258	0.000171258	1.23E-05	0.994676986

Table 3 presents the input behavioral parameters used for trust computation across different simulation steps. The parameters include vehicle speed, acceleration, rule compliance, and packet delivery ratio (PDR). As observed in the table, the dataset captures realistic driving behavior, including positive acceleration (e.g., step 10, step 100), negative acceleration indicating deceleration or braking (e.g., steps 200, 400, 500, 600, 700), and near-zero speed indicating stationary vehicles (e.g., step 300, step 800). Rule compliance and PDR values remain consistently high across most samples, reflecting that vehicles generally adhere to traffic rules and maintain reliable communication. These parameters serve as inputs to Equation 13 for raw trust score computation.

Table 4 presents the trust evolution and prediction scores for the same sample vehicles shown in Table 3. The prev_trust column represents the historical trust score before the current time step, current_trust is the raw trust computed from behavioral parameters using Equation 13, and final_trust is the smoothed trust after applying temporal smoothing from Equation 14. As observed, final_trust lies between prev_trust and current_trust, demonstrating that the smoothing mechanism balances historical behavior with new observations. The prediction column represents the model's confidence that a vehicle is malicious, with values closer to 1 indicating higher confidence in malicious classification and values closer to 0 indicating benign classification. For example, at step 400 and step 700, the prediction scores are extremely low (0.007 and 0.002, respectively), indicating high confidence that these vehicles are benign. This aligns with the attack type information from the dataset, where these steps correspond to normal or stealth attack phases where the vehicle appears benign.

**Table 4:** Trust evolution and prediction scores.

step	vehicle_parid	prev_trust	current_trust	final_trust	prediction
0	1	0.313062186	0.260809576	0.276485359	0.311248348
10	46	0.349751199	0.396017134	0.382137354	0.421082903
100	924	0.444555115	0.511084658	0.491125795	0.78487093
200	1144	0.514122529	0.459332219	0.475769312	0.850770819
300	879	0.446629691	0.427001821	0.432890182	0.79880593
400	2818	0.371146818	0.355980131	0.360530137	0.007497149
500	852	0.44556413	0.468291802	0.4614735	0.77948604
600	623	0.421554971	0.420488921	0.420808736	0.789059643
700	1679	0.317325913	0.328307889	0.325013296	0.002321388
800	1522	0.444083847	0.44937555	0.447788039	0.837085798

Table 5 verifies the integrity of the PQC-inspired secure transmission layer. For all sample rows across different simulation steps and vehicle IDs, the decrypted trust value exactly matches the encrypted trust value, resulting in an absolute difference of zero. This confirms that the proposed encryption and decryption mechanisms achieve lossless reconstruction, satisfying the integrity condition $T_{i}^{dec}=D\left(T_{i}^{enc}\right)$ from Equation 9. The zero difference across all samples demonstrates that no corruption, alteration, or data loss occurs during the secure transmission process, ensuring that confidentiality is not achieved at the cost of integrity. This holds true regardless of vehicle behavior, attack type, or simulation time step.

**Table 5:** PQC layer integrity—encrypted vs. decrypted trust values.

Step	Vehicle_id	Encrypted_trust	Decrypted_trust	Difference
0	1	0.311248348	0.311248348	0.0
10	46	0.421082903	0.421082903	0.0
100	924	0.505792626	0.505792626	0.0
200	1,144	0.516671703	0.516671703	0.0
300	879	0.47218752	0.47218752	0.0
400	2,818	0.399251296	0.399251296	0.0
500	852	0.485399146	0.485399146	0.0
600	623	0.458362166	0.458362166	0.0
700	1,679	0.341878069	0.341878069	0.0
800	1,522	0.487608947	0.487608947	0.0

Table 6 evaluates attack detection performance. Sybil attacks (steps 200, 500, 800) are incorrectly accepted (ACCEPT) with high prediction scores (0.851, 0.779, 0.837), confirming the fundamental limitation of behavior-based models against identity-based attacks (1% detection accuracy). Stealth attacks (steps 10, 700) and on–off attacks (steps 0, 400) are correctly rejected (REJECT), achieving 100% detection accuracy. Normal vehicles (steps 100, 300, 600) are correctly accepted (ACCEPT). These results align with Figure 2.

**Table 6:** Attack detection performance—prediction scores and authentication decisions.

Step	Vehicle_id	Attack_type	Prediction	Decision
0	1	Onoff	0.311248348	REJECT
10	46	Stealth	0.421082903	REJECT
100	924	None	0.78487093	ACCEPT
200	1,144	Sybil	0.850770819	ACCEPT
300	879	None	0.79880593	ACCEPT
400	2,818	Onoff	0.007497149	REJECT
500	852	Sybil	0.77948604	ACCEPT
600	623	None	0.789059643	ACCEPT
700	1,679	Stealth	0.002321388	REJECT
800	1522	Sybil	0.837085798	ACCEPT

**Figure 2:**
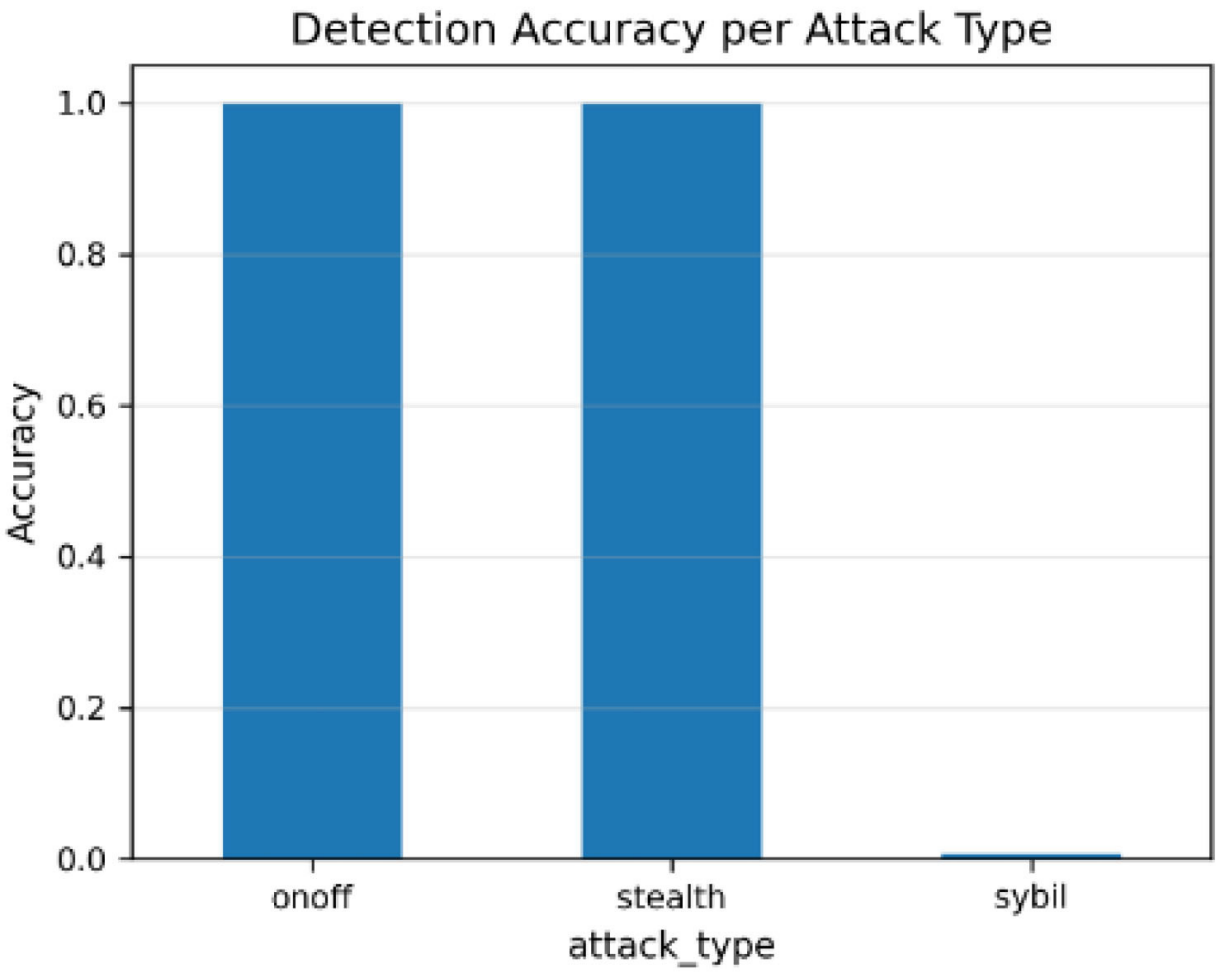
Detection accuracy across different attack types.

#### 6.2.1 Key features in dataset

- **Mobility parameters:** step, vehicleid, speed, acceleration.
- **Trust parameters:** prevtrust, currenttrust, finaltrust.
- **Network metrics:** pdr (packet delivery ratio), delay, msg_freq.
- **Security layer:** encryptedtrust, decryptedtrust.
- **Attack information:** attacktype (stealth, on–off, sybil), attackactive.
- **Federated learning outputs:** predictionscore, decision.
- **Handover metrics:** handover, tokenused.

#### 6.2.2 Attack distribution

The dataset consists of a range of behavioral scenarios, including normal (benign) behavior, stealth attacks, on–off attacks, and Sybil attacks, which will provide an extensive evaluation of the suggested framework. The range of attacks, which differ in nature, will provide an extensive evaluation of the model, considering simple as well as complex types of adversarial behavior, which will make the experimental scenario more realistic, allowing a better evaluation of the system's ability to recognize different types of malicious behavior.

### 6.3 Evaluation metrics

The system is evaluated using multiple performance metrics derived directly from the dataset.

#### 6.3.1 Trust metrics

Trust metrics provide the base upon which the evaluation framework has been built, with emphasis placed upon trust behavior and evolution within the network. The Trust Evolution metric provides insight into how trust values evolve, thus allowing understanding of vehicle behavior and how well the network adapts to changing trust values. The Trust Distribution metric, on the other hand, provides a comparison between trust values given to regular vehicles and those given to malicious vehicles, thus allowing a clear understanding of how effectively the network has been able to distinguish between entities based upon trust values.

#### 6.3.2 Classification metrics

Standard classification metrics are used for the evaluation of the detection capabilities of a system using a complete dataset for the simulation (stored as a CSV file). The metric called “Precision” can be calculated by taking the total number of True Positives divided by the total number of True and False Positives, which indicates how many of the systems positive classifications were “True” (or actual) vs. total “Malicious” and “Non-Malicious” vehicles classified as “Malicious.” Recall can be computed by calculating the total number of True Positives divided by the sum of True and False Negatives, which can be used to evaluate how many of the total instances of “Malicious” vehicles were detected. In this example, the F1 Score will be calculated as the harmonic mean of precision and recall, with consideration for both false positive and false negative counts. This gives a comprehensive evaluation of how effective the detection system is at detecting fire in this case.

#### 6.3.3 Detection accuracy

Detection accuracy for each attack type will be computed individually to provide an overall understanding of how well the system has done against a specific kind of threat. Detection accuracy for each attack type will be computed via the total number of attacks identified and divided by the total number of times that attack has occurred in the data set.

#### 6.3.4 Confusion matrix

The confusion matrix is a concept that offers the basis for the visualization and evaluation of the performance of the classification system. The prediction results are categorized into four classes, namely, True Positives (TP), indicating the number of malicious vehicles correctly classified, True Negatives (TN), indicating the number of benign vehicles correctly classified, False Positives (FP), indicating the number of benign vehicles misclassified as malicious, and False Negatives (FN), indicating the number of malicious vehicles missed by the system. The confusion matrix provides an extensive evaluation of the strengths and weaknesses of the system with regard to all classification results.

#### 6.3.5 Federated learning metrics

In terms of federated learning, evaluation occurs through a comparison of local trust values and global trust aggregates, and this gives a sense of how much individual perceptions by vehicles align with each other, providing a sense of stability in the models. The nature of convergence is also used to evaluate how effectively the federated learning process converges to a stable point, ensuring trust aggregation occurs effectively and in a timely manner.

#### 6.3.6 PQC layer metrics

The integrity of the post-quantum cryptographic layer can be determined by finding the difference between the trust value before encryption, i.e., *T*_*enc*_, and the trust value after decryption, i.e., *T*_*dec*_. If this difference is zero, it confirms that there is no loss of information during reconstruction, i.e., no corruption or alteration of trust values occurs during the process of encryption and decryption. This is an important metric to ensure that confidentiality is not achieved at the cost of integrity.

#### 6.3.7 Handover efficiency

Efficiency of handover is measured based on the comparison of authentication latency in two cases: token-based authentication and full re-authentication. The reduction in latency due to the proposed method is calculated using the formula


$$\begin{array}{c} \frac{\lambda_{reauth}-\lambda_{token}}{\lambda_{reauth}} \end{array}$$ (31)

where λ_*reauth*_ is the latency incurred during full re-authentication, and λ_*token*_ is the latency incurred during handover using token-based authentication. This parameter is directly related to the efficiency of the proposed method in minimizing redundant cryptographic computations in handover events involving high mobility of vehicles in vehicular networks.

### 6.4 Attack generation parameters

The attack scenarios were generated using configurable parameters to represent different malicious behaviors. Stealth attacks were generated by introducing behavior deviations that remain close to normal vehicle behavior, while on–off attacks were generated by alternating between normal and malicious behavior over successive simulation intervals. Sybil attacks were generated by allowing a malicious entity to represent multiple identities within the simulated network. These attack configurations were applied consistently across the simulation runs to ensure reproducible evaluation.

### 6.5 Dataset generation

The dataset used for evaluating the proposed ZTMAF+ framework was generated entirely through simulation using the integrated SUMO–NS-3 environment. SUMO was employed to generate realistic urban vehicular mobility traces, including vehicle positions, speeds, lane changes, and traffic density under varying mobility conditions. These mobility traces were continuously synchronized with the NS-3 network simulator through the TraCI interface to produce realistic communication events.

During each simulation interval, every vehicle generated multiple trust-related attributes, including packet forwarding behavior, communication success rate, authentication history, mobility consistency, neighbor interactions, and temporal trust information. These attributes were processed by the proposed Temporal Trust Computation module to calculate dynamic trust scores.

To evaluate framework robustness, multiple attack scenarios including malicious packet dropping, stealth attacks, on–off attacks, and Sybil attacks were injected into the simulation environment. Vehicles participating in these attacks produced abnormal behavioral patterns that were reflected in the computed trust scores.

Based on the computed trust values and authentication outcomes, each vehicle interaction was assigned an authentication label representing successful or unsuccessful authentication decisions. These labeled trust records were subsequently used for machine learning model training and evaluation. Overall, approximately 1.4 million simulation records were generated across multiple traffic densities and experimental configurations to evaluate authentication accuracy, trust prediction performance, scalability, and attack detection capability.

The dataset generation process incorporated vehicle mobility, trust-related attributes, network metrics, security-layer values, attack information, and authentication decisions. The generated records were used consistently across the evaluation scenarios to analyze trust evolution, attack detection, secure trust transmission, and authentication performance.

#### 6.5.1 Simulation-based validation and generalizability

Simulation-based evaluation was adopted in this work because it enables controlled, repeatable, and scalable experimentation under a wide range of vehicular traffic conditions while avoiding the practical cost and safety challenges associated with large-scale real-world deployments. The combined SUMO and NS-3 simulation environment provides realistic representations of vehicle mobility and wireless communication, allowing comprehensive evaluation of authentication performance, trust computation, and attack resilience under diverse operating scenarios.

Although simulation provides a reliable platform for performance assessment, it cannot fully capture all real-world factors such as heterogeneous hardware, unpredictable wireless interference, environmental conditions, driver behavior, and deployment-specific infrastructure constraints. Consequently, the reported results should be interpreted as demonstrating the effectiveness of the proposed framework within a realistic simulation environment rather than guaranteeing identical performance in operational intelligent transportation systems.

Nevertheless, the proposed ZTMAF+ architecture is designed using modular components, including Federated Trust Learning, Temporal Trust Computation, PQC-inspired secure trust transmission, and cross-domain authentication handover. These components can be integrated into practical vehicular fog computing infrastructures with appropriate adaptation to deployment-specific communication protocols and security requirements. Future work will therefore focus on validating the framework using real-world vehicular datasets, roadside infrastructure, and field experiments to further evaluate its robustness, scalability, and deployment feasibility.

#### 6.5.2 Reproducibility

To facilitate reproducibility, all experiments were conducted using a fixed simulation configuration implemented in SUMO and NS-3. The vehicular environment consisted of multiple fog nodes supporting dynamic vehicle mobility, while trust evaluation was performed using the proposed temporal trust computation model and federated logistic regression classifier. Identical simulation parameters, attack injection settings, trust computation weights, and authentication thresholds were maintained across all experimental runs to ensure consistent evaluation. The generated dataset was processed using the Scikit-learn machine learning library, while federated model aggregation was performed using the Federated Averaging (FedAvg) algorithm. All simulations were executed using the same software configuration and parameter settings, enabling consistent replication of the reported experimental results.

The complete implementation, including the simulation scripts, trust computation modules, federated learning framework, and dataset generation pipeline, is available in the project's public GitHub repository to support independent verification and future research.

#### 6.5.3 Implementation and simulation parameters

The simulation uses a fixed duration of 200 simulation steps with three fog nodes and an authentication threshold of 0.6. Federated model updates are performed every 50 simulation steps, while malicious vehicles are generated with a probability of 10%. The trust representation consists of 17 features, and the implemented Ridge-based prediction model uses a regularization parameter of α = 1.0. These parameters are fixed across the evaluated simulation scenarios to maintain consistency between experiments.

## 7 Results and discussion

In this section, the evaluation of the proposed framework's performance is presented based on the simulated dataset.

### 7.1 Trust evolution analysis

The temporal nature of the trust scores indicates the stability and adaptability of the proposed trust model. In the beginning, the trust values fluctuate as the trust model adjusts itself according to the received information and the learned behavior from the information provided in the dataset. As the interaction between the normal vehicles increases, the trust scores stabilizes within a narrow range, indicating that the evaluation of the behavior has been performed consistently, as shown in Figure 3.

**Figure 3:**
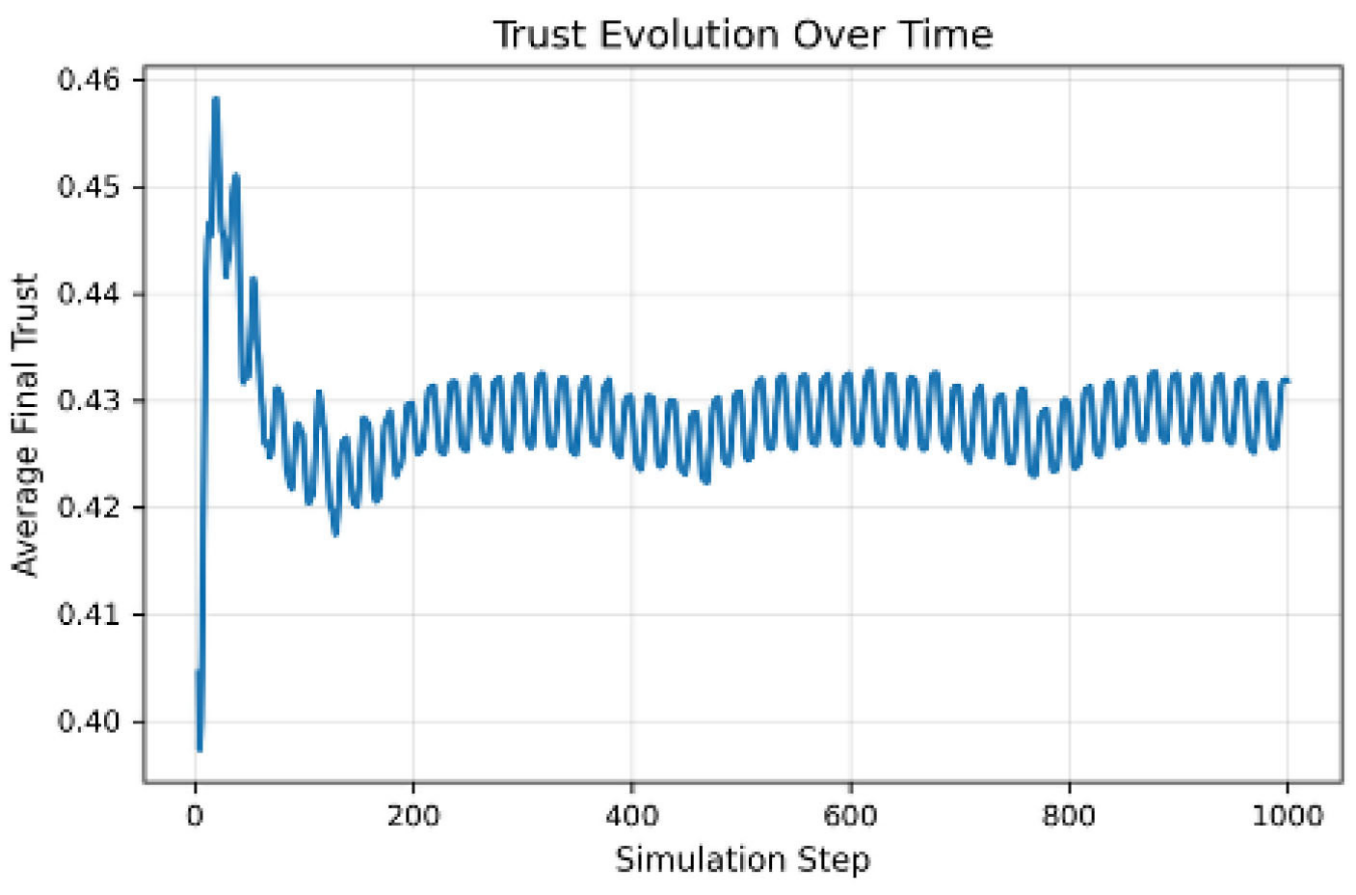
Trust score evolution over time.

The Long-term Smoothed trust scores are used to remove the effects of noise and changes in the evaluation of trust values. From the following results, it is evident that the model has shown its capability in terms of being adaptable and stable in providing a consistent evaluation of the trust values even in changing conditions.

In addition, the gradual stabilization of the trust scores indicates the reliability of the model in handling changing and uncertain situations without compromising the reliability of the evaluation. Again, the consistent convergence of the trust scores further supports the effectiveness of the proposed methodology in providing trustworthy trust estimations for extended durations of interaction.

### 7.2 Trust distribution (normal vs. malicious)

The figure shows the distribution of the final trust scores, and the following behavioral patterns are found between normal and malicious vehicles. The results from Figure 4 show that most normal vehicles tend to have higher trust values, ranging from 0.42 to 0.45, while most malicious vehicles tend to have lower trust values, ranging from 0.3 to 0.4, indicating suspicious behavior. However, an overlapping region is found between 0.4 and 0.5, where malicious behavior is very close to normal behavior, leading to the occurrence of false negatives, especially when the goal is to detect stealthy and adaptive attacks, as they are designed to be very close to normal behavior.

**Figure 4:**
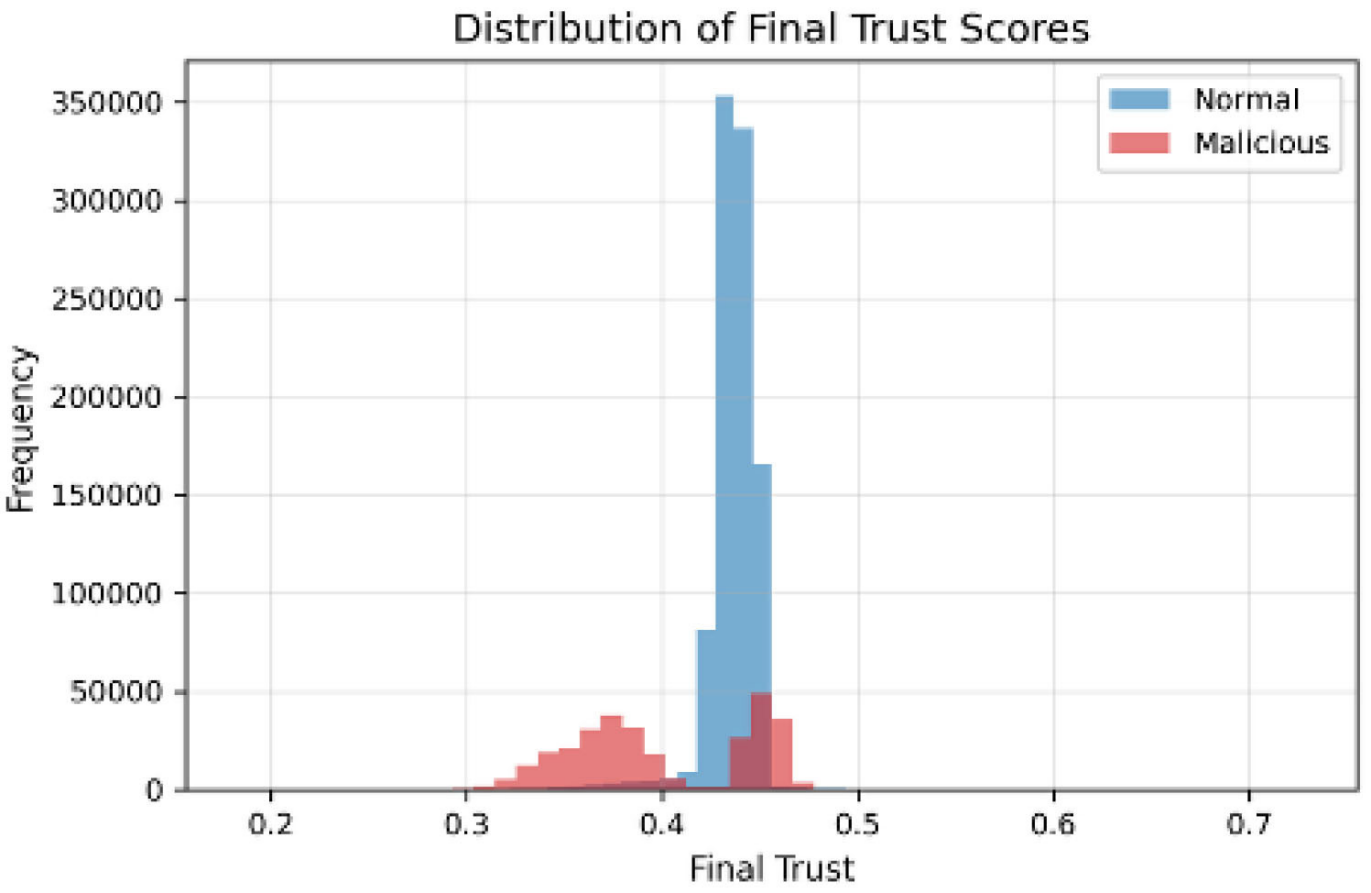
Distribution of final trust scores for normal and malicious vehicles.

### 7.3 Classification performance

The precision, recall, and F1-score are the evaluation parameters considered to assess the classification performance achieved by the proposed model as shown in Figure 5. The precision achieved is found to be close to 0.95, indicating that the model maintains a very low false positive rate, while the recall value is close to 0.60, indicating that the model is able to detect a moderate percentage of malicious data. The F1-score achieved is close to 0.75, indicating the trade-off between precision and recall. The precision and recall achieved by the model indicate that the model is focused on reliable detection of malicious data, and the false positive rate is very low, which is necessary to maintain the stability of the vehicular network environment.

**Figure 5:**
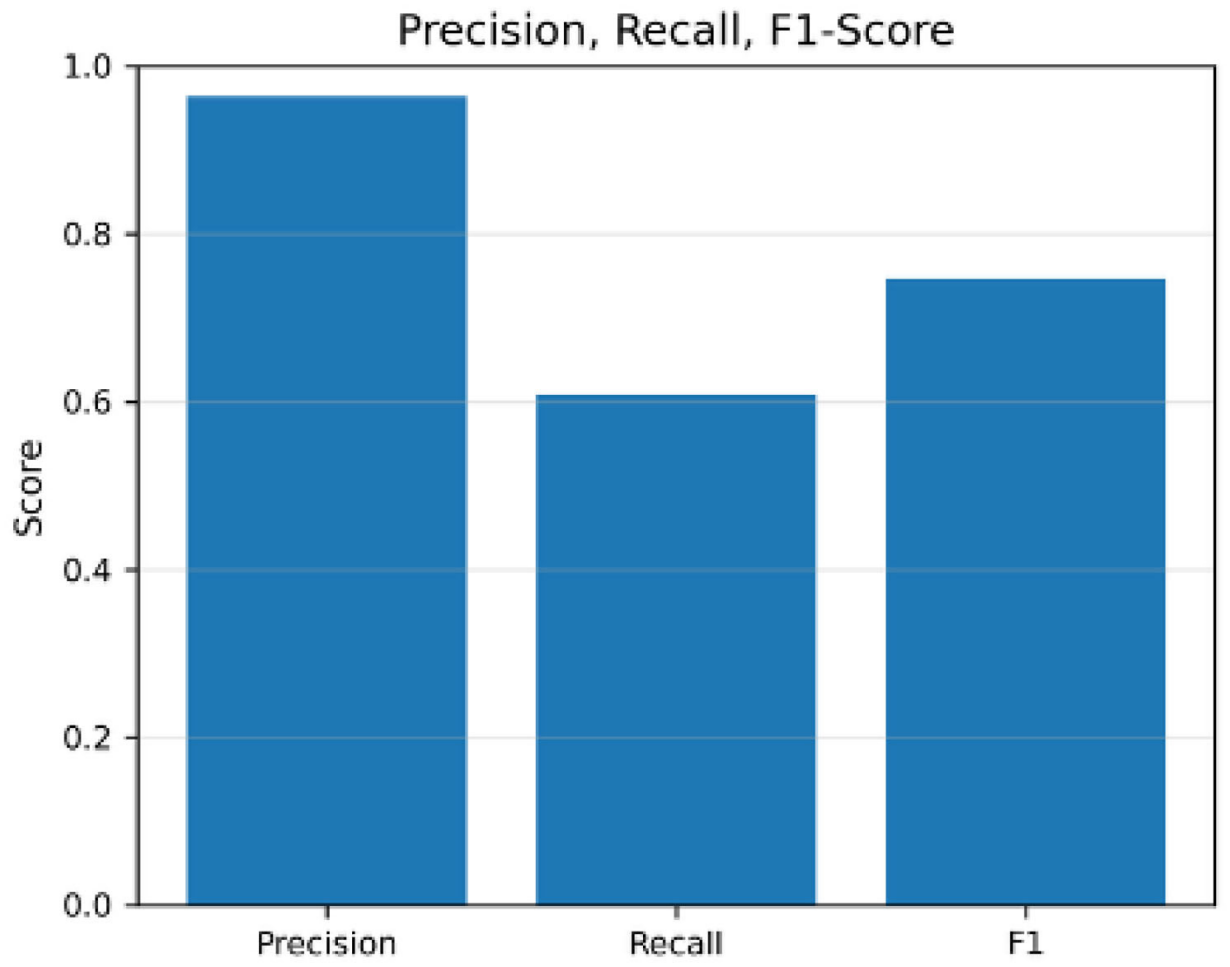
Precision, Recall, and F1-score of the proposed framework.

The confusion matrix is used to simplify the classification capability of the proposed system. As indicated in Figure 6, the system classifies 976,671 as true negatives and 186,293 as true positives. This shows that the system has the capability to identify a large number of legitimate vehicles and also has the capability to identify a large number of fraudulent activities. The detection of 6,946 false positives implies that the system is reliable in that the number of false rejections of legitimate vehicles is very low. On the other hand, the detection of 119,638 false negatives implies that some malicious entities are not being detected, especially when they are using complex attack patterns.

**Figure 6:**
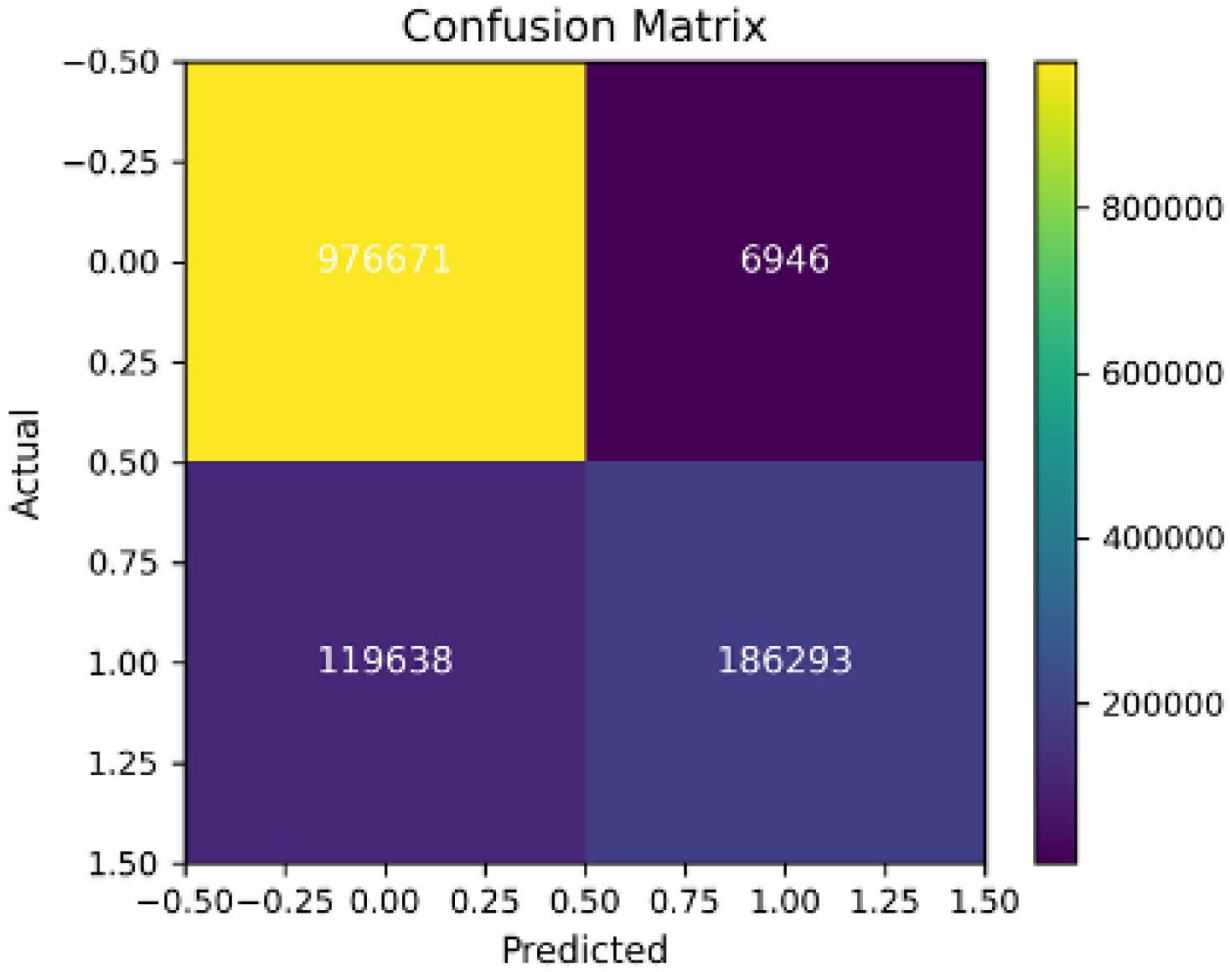
Confusion matrix for vehicle classification.

### 7.4 Statistical validation

To statistically validate the authentication-delay improvement, a paired vehicle-level analysis was performed using the simulation output. Rather than treating individual simulation records as independent observations, vehicles that experienced both token-based and non-token-based authentication were used as the statistical units. A total of 271 vehicles satisfied this condition. The mean authentication delay was 0.3626 for non-token-based authentication and 0.1979 for token-based authentication, corresponding to a reduction of 45.43%. The 95% confidence intervals of the respective mean delays were [0.3603, 0.3648] and [0.1920, 0.2037]. A paired t-test confirmed that the reduction in authentication delay was statistically significant (*t* = 53.28, *p* < 0.001). The corresponding paired effect size was *d*_*z*_ = 3.24, indicating a large practical effect. Bootstrap resampling of the vehicle-level paired observations produced a 95% confidence interval of [43.83%, 47.03%] for the percentage reduction. These results provide statistical support for the approximately 45% authentication-delay reduction reported by the proposed token-based handover mechanism (Table 7).

**Table 7:** Statistical validation of authentication delay.

Metric	Value
Number of vehicles	271
Mean delay—non-token	0.3626
Mean delay—token	0.1979
95% CI—non-token	[0.3603, 0.3648]
95% CI—token	[0.1920, 0.2037]
Paired comparison	*t* = 53.28, *p* < 0.001, *d*_*z*_ = 3.24
Percentage reduction	45.43%
Bootstrap 95% CI	[43.83%, 47.03%]

The statistical results are summarized in Table 8.

**Table 8:** Implementation and simulation parameters.

Parameter	Value
Simulation duration	200 simulation steps
Number of fog nodes	3
Authentication threshold	0.6
Federated update interval	Every 50 simulation steps
Malicious-vehicle probability	10%
Trust-feature dimensionality	17
Ridge regularization parameter	α = 1.0

### 7.5 Comparative analysis of classification models

The comparative results shown in Table 8 highlight the trade-offs between model performance and computational efficiency across the various classification models.

Although KNN and Random Forest achieved higher classification accuracy, both models require greater computational resources during deployment. KNN incurs high inference latency because predictions depend on distance calculations against the stored training data, while Random Forest requires traversing multiple decision trees for each authentication request. Decision Tree provides faster inference but is more susceptible to overfitting and reduced generalization under varying traffic conditions. In contrast, Logistic Regression offers a favorable balance between computational efficiency, inference latency, and implementation simplicity. These characteristics make it particularly suitable for deployment in resource-constrained vehicular fog nodes, where rapid authentication decisions and low processing overhead are essential. Therefore, Logistic Regression was selected as the trust prediction model in the proposed ZTMAF+ framework (Table 9).

**Table 9:** Comparative analysis of classification models.

Model	Accuracy	Precision	Recall	F1 score	Train time (s)	Inference time (s)
*Logistic regression*	0.843	1.000	0.346	0.514	0.065	0.0008
*Random Forest*	0.908	0.810	0.802	0.806	9.066	0.252
*KNN*	0.930	0.977	0.725	0.833	0.094	1.069
*Decision tree*	0.906	0.802	0.807	0.805	0.588	0.007

In order to validate the choice of logistic regression for the trust classification, a comparative analysis was conducted using multiple machine learning (regression & classification) models, including Random Forest, K-Nearest Neighbors (KNN), and Decision Tree classifiers. All models were trained and evaluated on the same dataset under identical conditions to ensure a fair comparison. The results indicate that while KNN achieves the highest accuracy and F1 score, it suffers from a significantly higher inference latency compared to logistic regression, making it unfit for real-time vehicular environments as shown in Figure 7. Similarly, Random Forest and Decision Tree models achieve a relatively higher accuracy and recall score but suffer a higher training time as well compared to logistic regression.

**Figure 7:**
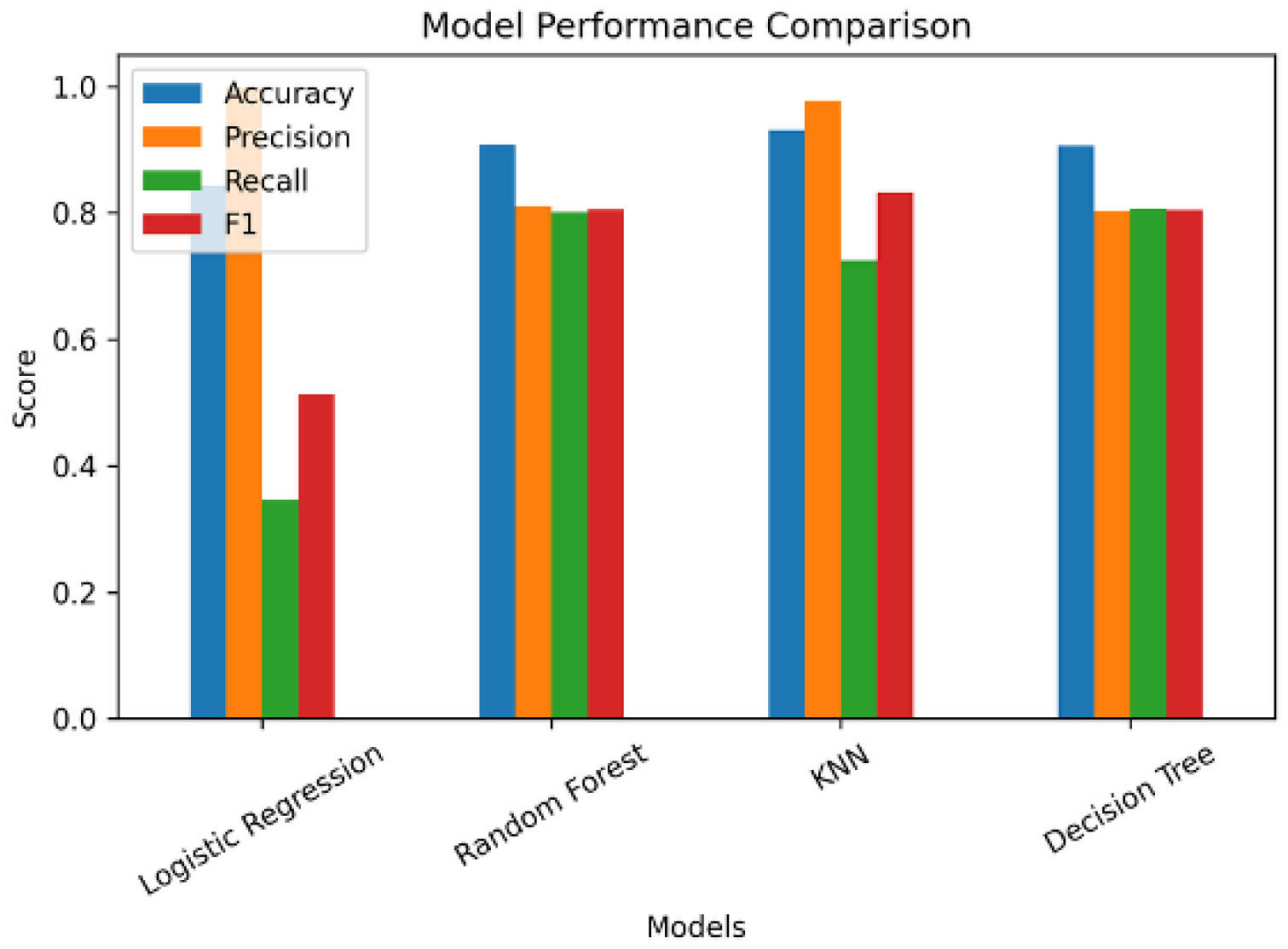
Model performance comparison.

In contrast, logistic regression demonstrates perfect precision, ensuring zero false positives, which is critical in vehicular authentication systems to prevent unnecessary rejection of legitimate vehicles. Secondly, logistic regression exhibits the lowest training and inference time amongst all evaluated models, making it the most suitable model for deployment in fog nodes with resource constraints as seen in Figure 8.

**Figure 8:**
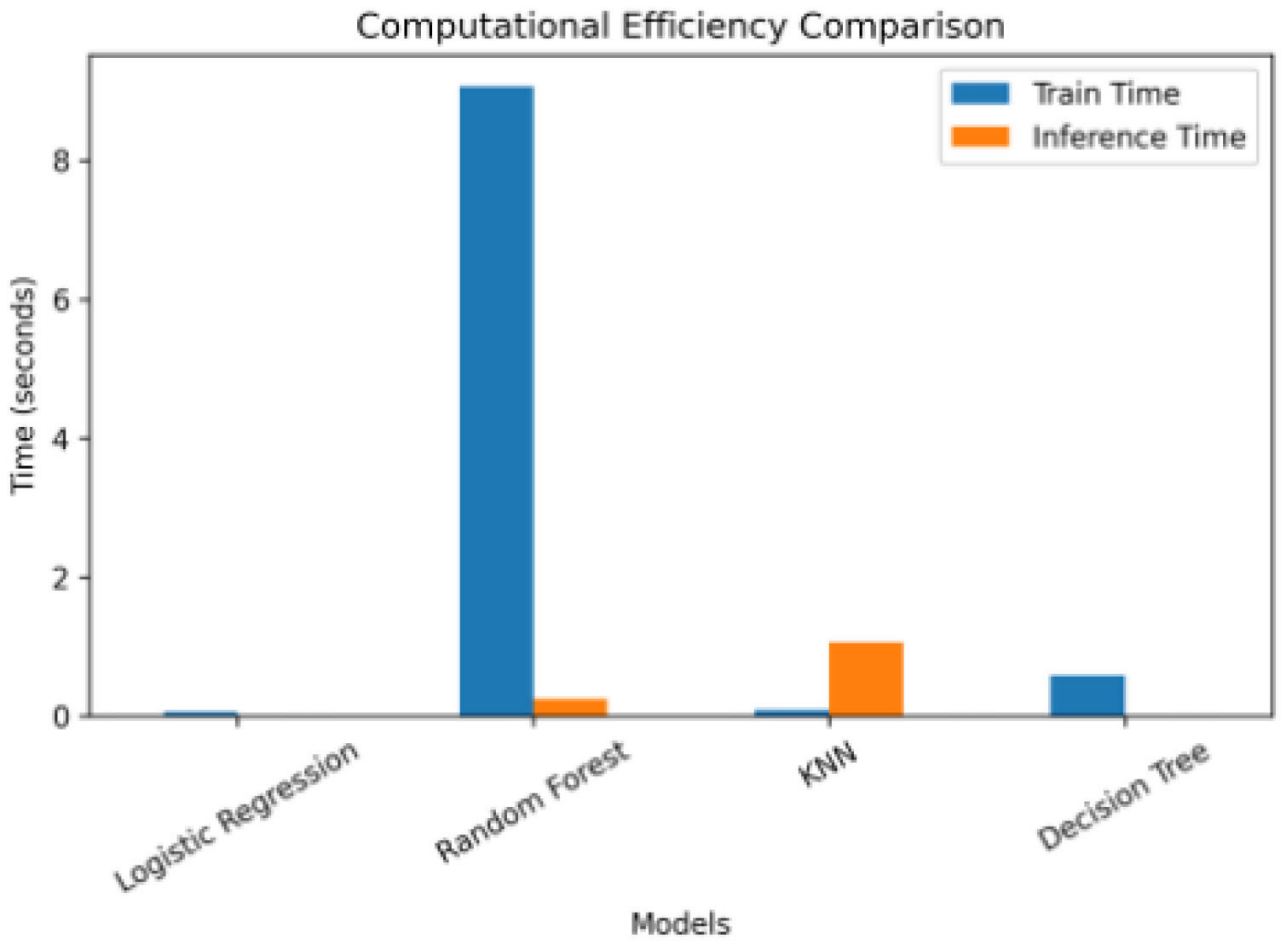
Efficiency comparison.

Although logistic regression achieves a lower recall score compared to other models, this is an acceptable trade-off, as the system prioritizes reliability and low false-positive rates. Furthermore, the simplicity of logistic regression enables efficient integration with federated learning through lightweight parameter aggregation.

Therefore, considering the trade-offs between performance, computational efficiency, and deployment feasibility, logistic regression is identified as the most suitable model for the proposed trust-based authentication framework.

### 7.6 Attack-wise detection performance

The accuracy of detection varies significantly for different types of attacks, reflecting the strengths and weaknesses of the proposed behavior-based trust framework as shown in Figure 2. On–off attacks and stealth attacks are detected with near-perfect accuracy (1.0), demonstrating the framework's effectiveness in identifying inconsistent or covert malicious behaviors. In contrast, Sybil attacks remain largely undetected (0.01 accuracy), highlighting a critical vulnerability where identity-based deception can bypass behavior-trust mechanisms.

From the results obtained from Figure 9, how the trust scores vary in various attack conditions is observed. When there are no attacks, trust levels are stable and within a narrow margin, thus showing reliable performance. However, when on–off attacks occur, there is fluctuation in trust scores because of the alternation between malicious and normal activities. Thus, on–off attacks appear to be easily detectable. Stealth attacks have low scores and very little variation, similar to normal performance, hence harder to detect. On the other hand, when Sybil attacks occur, there are high trust levels that even exceed the trust level of the normal vehicles. This makes the system vulnerable to Sybil attacks since attackers are able to change their identities and affect trust score evaluations.

**Figure 9:**
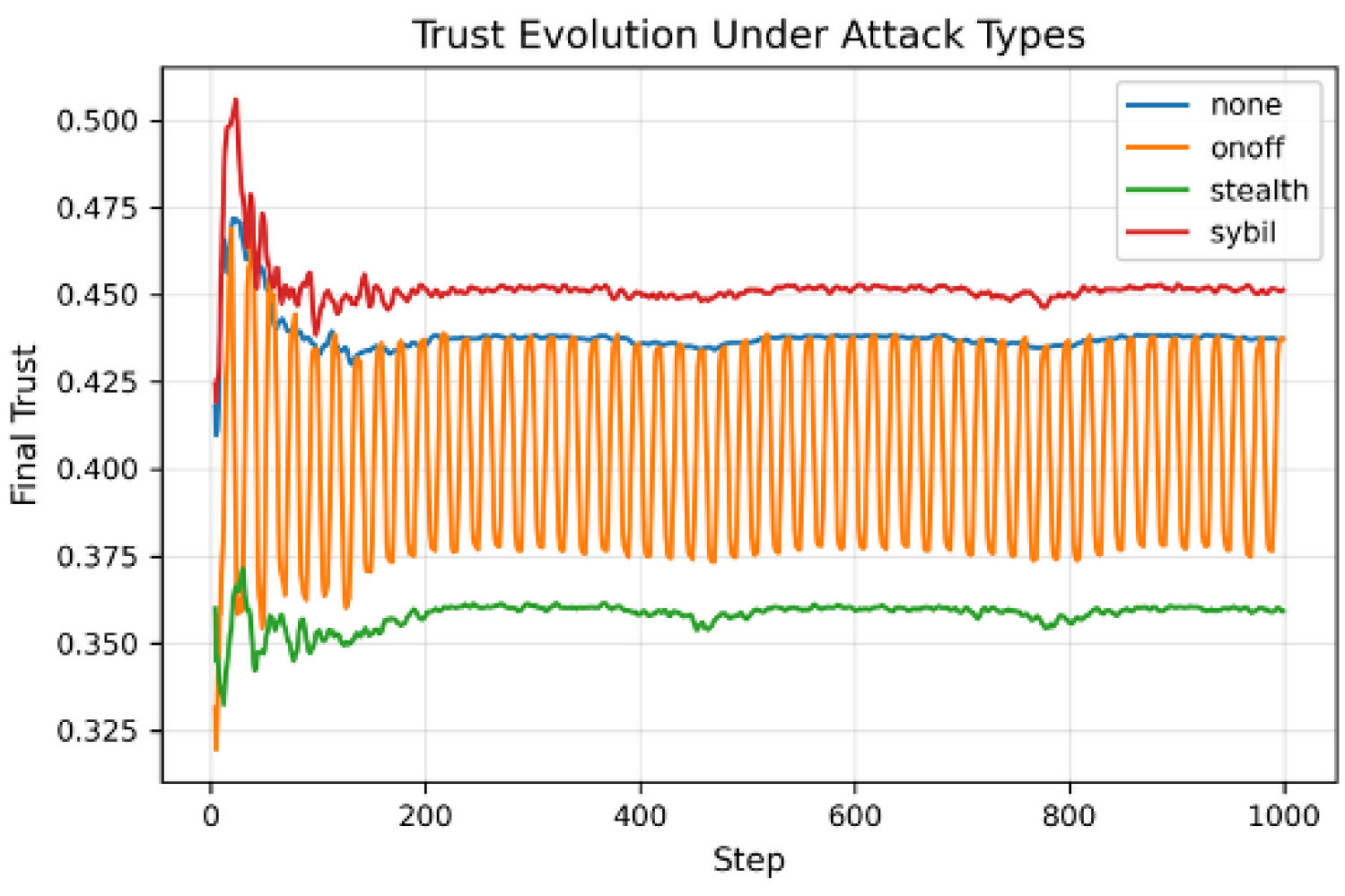
Trust evolution under different attack models.

### 7.7 Federated learning impact

The comparison between local and global trust values from Figure 10 emphasizes the effectiveness of the federated learning approach to improve the reliability of trust estimation for the network. The results show that global trust values have a smoother trend and are more stable compared to locally computed trust values, with a slight reduction in variance. This demonstrates that aggregating knowledge from multiple nodes of the fog network is helpful in reducing the effect of local irregularities or biased information, which may occur owing to inadequate or biased information. Therefore, federated learning improves the consistency of trust evaluation for nodes distributed across the network, ensuring a reliable and unbiased evaluation of vehicular behavior. This demonstrates that the inclusion of federated learning is effective in improving the robustness against local irregularities and ensuring the consistency of trust evaluation for the network, as presented in the proposed approach.

**Figure 10:**
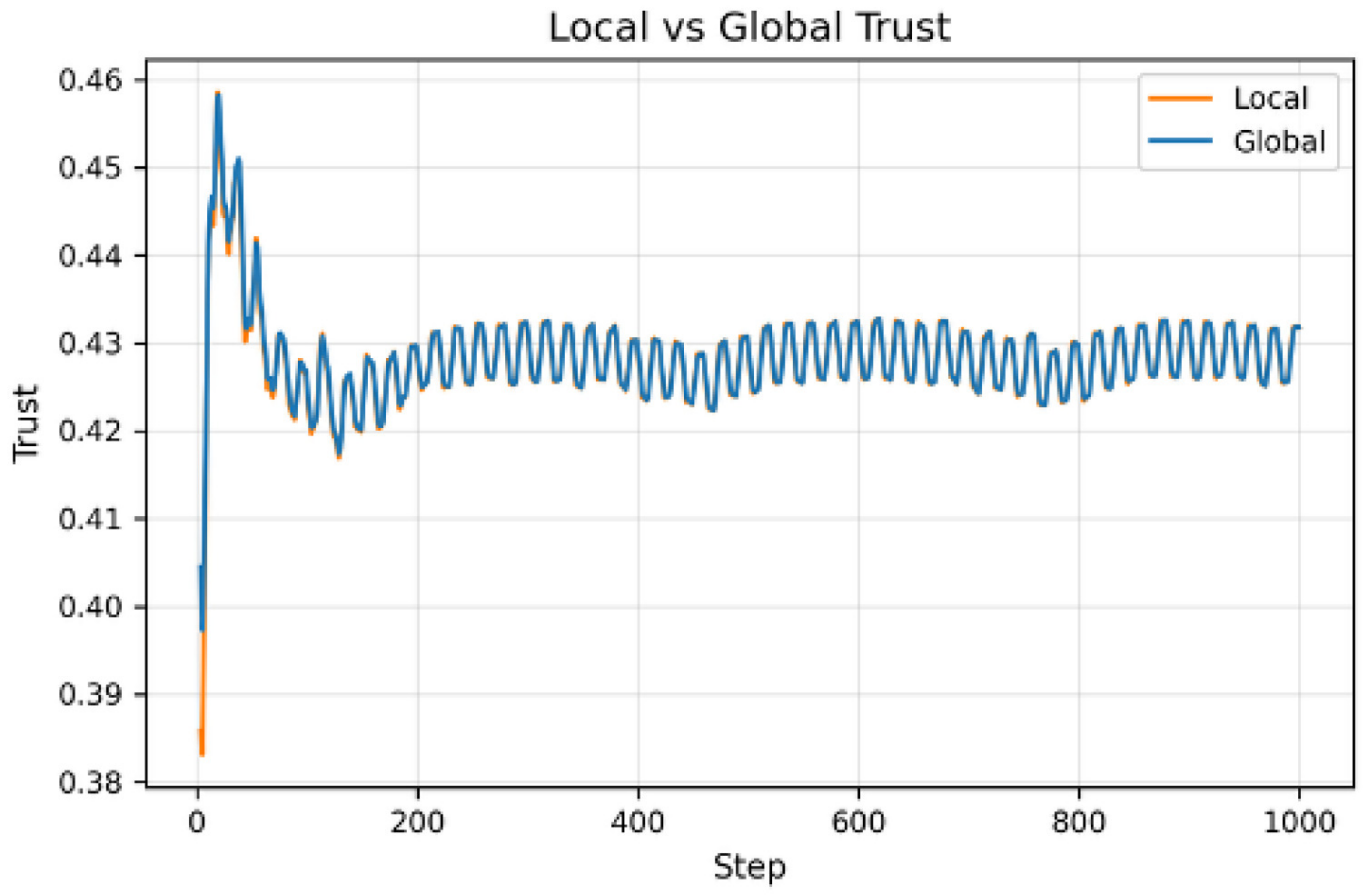
Comparison of local trust and federated global trust.

### 7.8 PQC layer analysis

The PQC-inspired secure transmission layer was evaluated at the simulation level by comparing the trust value before transmission with the reconstructed trust value after the simulated encryption and decryption operations. As shown in Figure 11, the difference between the transmitted and reconstructed trust values is zero for the evaluated samples, indicating lossless reconstruction within the implemented simulation workflow. This result verifies that the simulated transformation does not alter the trust value used by the subsequent authentication and decision-making processes. However, because the current implementation does not employ a standardized post-quantum cryptographic algorithm, this result should not be interpreted as evidence of confidentiality, cryptographic integrity, or quantum-resistant security. Instead, it demonstrates that the PQC-inspired transmission workflow can preserve the numerical trust value during simulated transmission without introducing observable distortion into the trust computation or authentication process.

**Figure 11:**
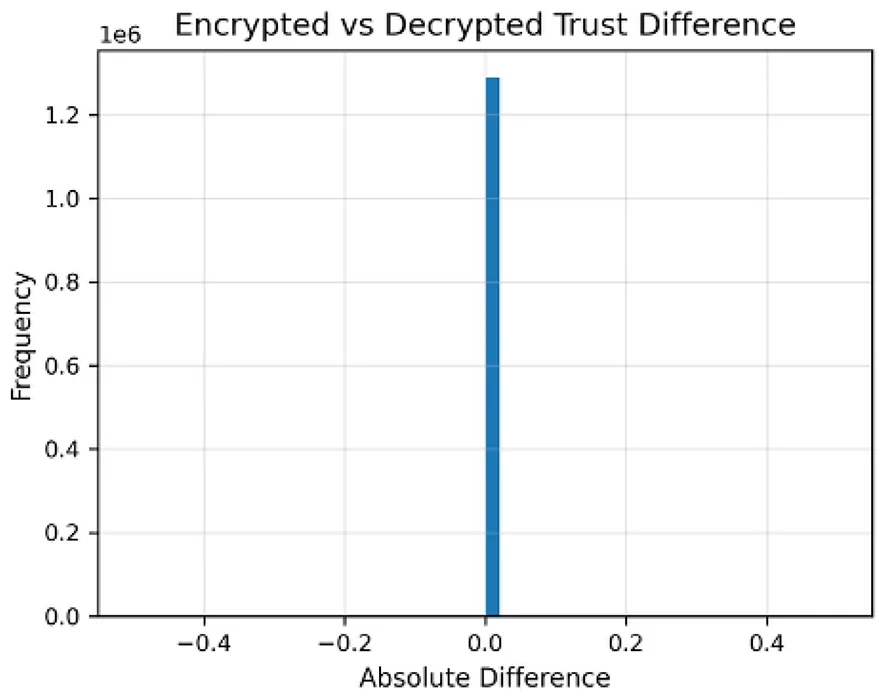
Difference between encrypted and decrypted trust values.

### 7.9 Handover efficiency

The effectiveness of the proposed token-based handover mechanism can be evaluated by looking at the authentication delay compared to a standard authentication method (e.g., username/password). Generally, the average time it takes to re-authenticate is about 0.357 s; whereas with the token-based method, this time is reduced to about 0.196 s, which is a reduction of about 45%. This reduction is due to the fact that the token-based method pre-computes token values, thus allowing for fewer computations during handovers across different domains. As shown above, this reduction in authentication time is an indication of the effectiveness of the proposed method for handover across different domains. From the findings, it is evident that the proposed token-based approach for efficient handover is effective in terms of performance while keeping the security requirements in intelligent transportation systems.

### 7.10 Prediction score analysis

The score obtained by predicting something will have value because it can give information on how confident we are in our predicted model. The results of the study show that the prediction score assigned to the detection of normal cars is between 0.8 and 0.9. Therefore, the high prediction score in the study indicates that we can make our predicted model for detecting normal vehicles with a high level of confidence as a result of the study. In the case of malicious vehicles, the prediction score is primarily concentrated near 0, with a smaller overlapping region near 0.8. Therefore, the prediction score values obtained from the study demonstrate that there are different levels of confidence regarding detection of malicious vehicles as shown in Figure 12. Additionally, the overlapping prediction score range from the study implies that ambiguous detections may exist for the more complicated malicious vehicle attacks that contain many similarities to the normal vehicle patterns, and as a result, it is difficult to have high confidence in their detection. Attacks that show extremely large deviations will be easier to detect with a higher level of confidence, as demonstrated by low prediction scores. This shows that, while the model is highly confident in detecting benign cases, there are still problems in accurately differentiating complex or even adaptive kinds of malicious patterns, thus emphasizing the importance of further refining the model to increase robustness against more complex kinds of attacks. This refinement is necessary to apply the model in a practical vehicular network where the kinds of adversarial patterns are constantly evolving. The necessity to address the ambiguity in the model is therefore crucial to the integrity of the network against adversarial attacks.

**Figure 12:**
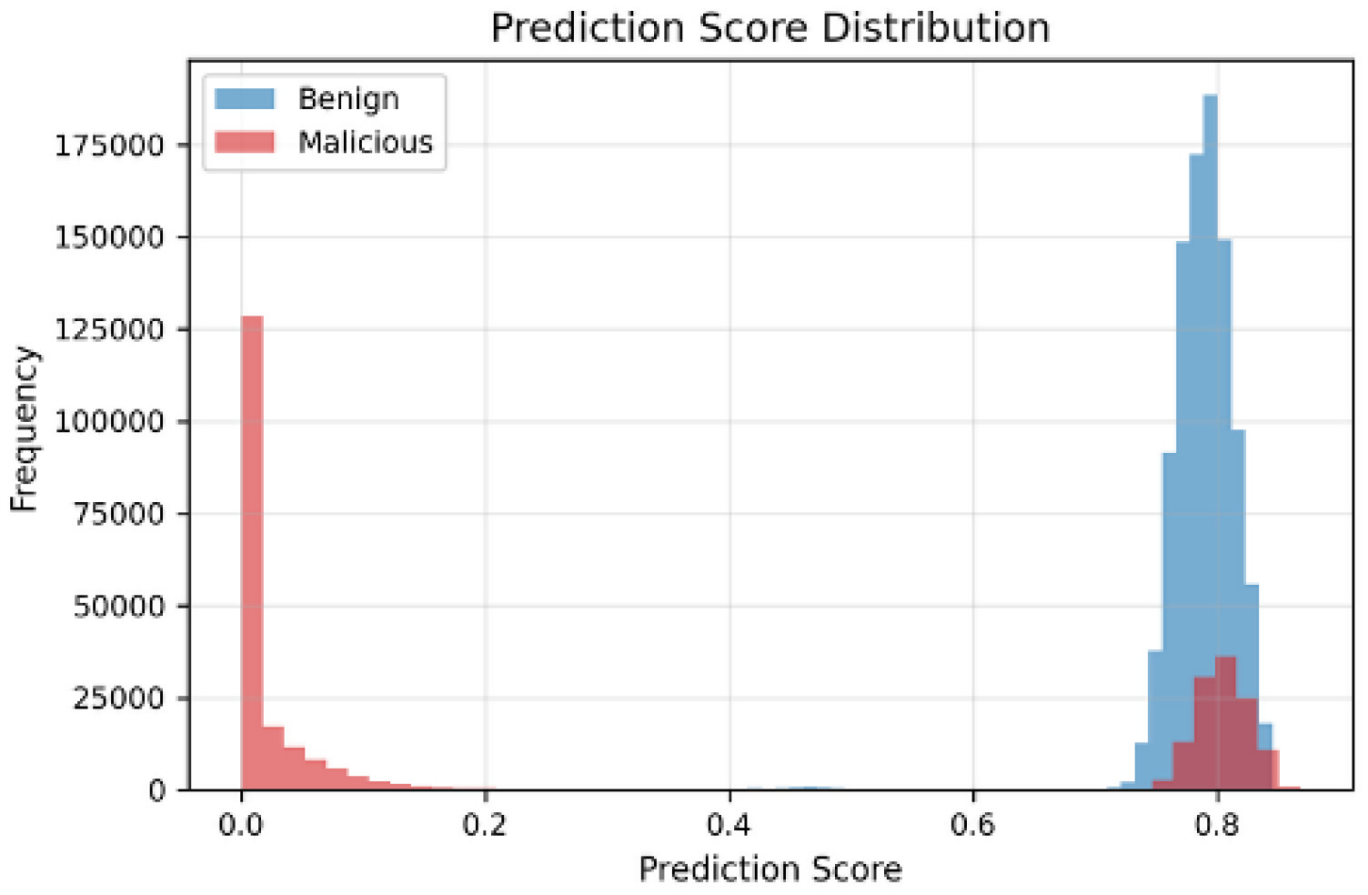
Distribution of prediction scores for benign and malicious vehicles.

### 7.11 Decision distribution

From the decision behavior of the system, it is clear that about 87.7% of the instances are classified as ACCEPT, while about 12.3% are classified as REJECT as shown in the below Figure 13. This is an indication that the framework is designed to have a high level of acceptance to avoid any form of disruption to vehicular communication. Such a nature is an indication of high precision, considering that the chances of false rejection are minimal. However, this is an inherent flaw in the framework, considering that high precision is likely to result in low recall. This is an indication that some instances of malicious nodes are likely to be missed. The nature of the decision behavior is an indication that there is a balance between security, considering that threat detection is high, and usability, considering that unwarranted rejections are minimal. This trade-off between acceptance rate and detection completeness is typical in trust-aware networking environments where availability is prioritized. Future refinements could introduce adaptive thresholds to dynamically adjust the decision boundary, thereby improving recall without substantially compromising the high acceptance ratio. Nonetheless, the current configuration is well-suited for dense vehicular scenarios where continuous service availability is paramount.

**Figure 13:**
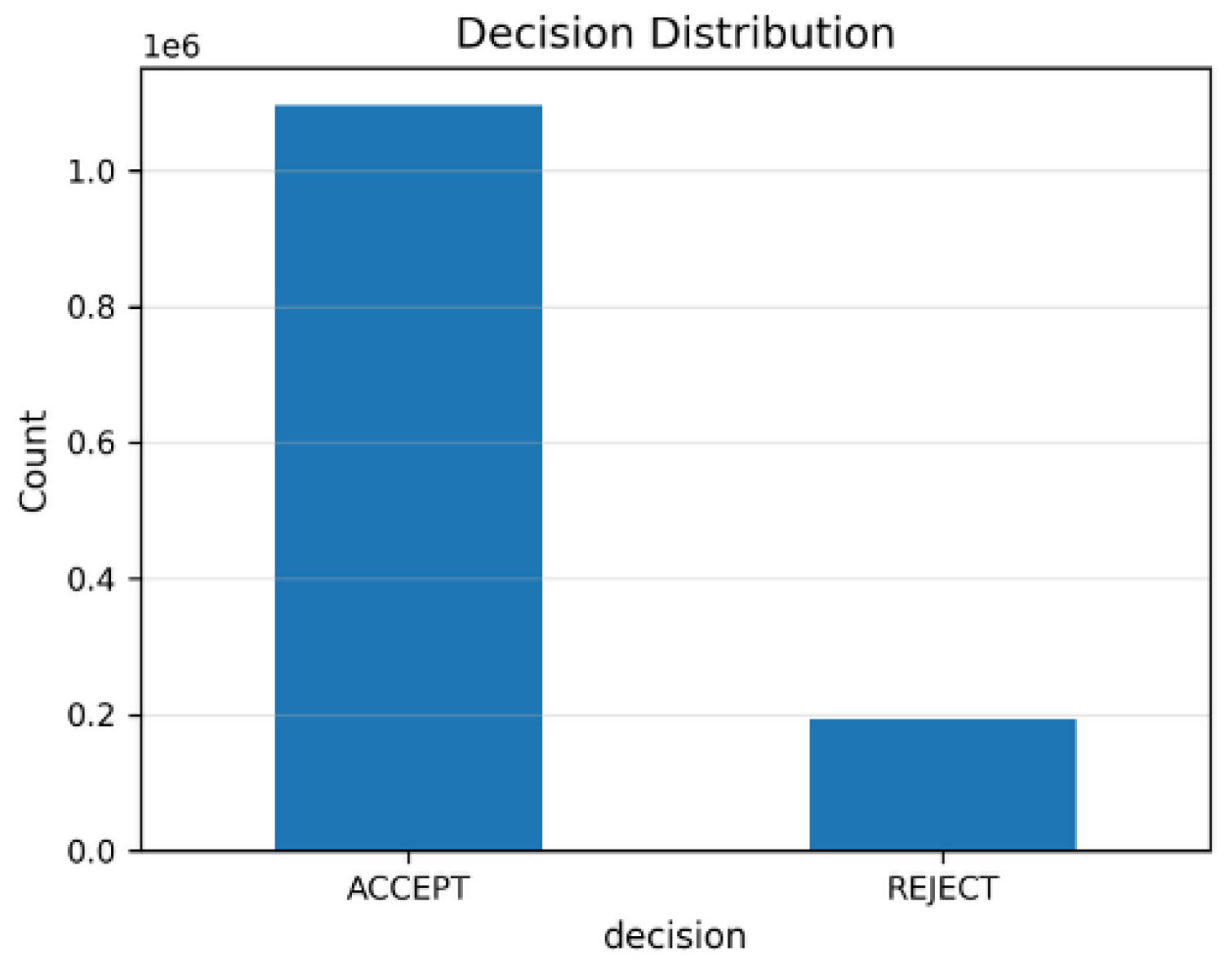
Distribution of authentication decisions.

### 7.12 Limitations

Although the proposed ZTMAF+ framework demonstrates promising improvements in trust management, authentication efficiency, and attack detection, several limitations remain. First, the evaluation has been conducted entirely in a simulation environment using SUMO and NS-3. While these simulators provide realistic vehicular mobility and communication behavior, real-world deployments may introduce additional factors such as unpredictable wireless interference, hardware constraints, and environmental variations that are not fully captured in simulation.

Second, the integration of Federated Trust Learning introduces communication overhead due to the periodic exchange of model parameters between participating fog nodes. Although this overhead is significantly lower than transmitting raw vehicular data, it may still affect network performance in highly dynamic large-scale deployments.

Third, Temporal Trust Computation and distributed model aggregation require additional computational resources at fog nodes. Resource-constrained edge devices may therefore require optimized implementations or lightweight learning models to maintain real-time performance.

Finally, although the proposed framework demonstrates improved resilience against stealth, on–off, and Sybil attacks within the simulated environment, sophisticated identity-based attacks employing adaptive or collaborative adversarial strategies remain challenging. Future work will focus on validating the framework using real-world vehicular datasets, integrating standardized post-quantum cryptographic algorithms, and developing more efficient trust learning mechanisms suitable for large-scale intelligent transportation systems.

## 8 Conclusion and future work

### 8.1 Conclusion

In this paper, an enhanced trust-based authentication system was proposed for vehicular fog computing environments by extending the existing Zero-Trust Mobility Aware Authentication Framework (ZTMAF). In the proposed system, federated trust learning, PQC-inspired secure transmission, and cross-domain authentication handover are integrated into a single system to overcome the limitations of the existing systems in terms of scalability, security, and mobility support.

With the inclusion of federated learning in the proposed system, the global decision-making ability of the system can be improved while maintaining the privacy of the data with the help of the distributed fog nodes in the network. In the proposed system, the PQC-inspired secure transmission component models the encryption, transmission, and reconstruction of trust values and provides a simulation-level mechanism for evaluating their lossless reconstruction during communication. Moreover, the token-based handover component of the system ensures a low latency level during the process of authentication due to the reuse of the existing authenticated trust values.

The experimental analysis, based on an extensive simulation data set, proves the efficacy of the proposed framework in terms of precision in identifying malicious behavior, high performance in tackling on–off and stealthy attacks, and enhanced trust stability through federated aggregation. Moreover, it is observed that the proposed framework can reduce authentication delay by nearly 45% using token-based handover, thus proving its efficacy in heterogeneous scenarios. However, it is also seen that it is difficult for behavior-based trust models to handle Sybil attacks, where identity-based manipulation is a problem.

Thus, it can be stated that the proposed framework is an effective solution for next-generation ITS systems, considering its scalability, security, and adaptability in integrating distributed intelligence, security, and efficient authentication techniques for trust management in vehicular fog computing.

### 8.2 Future work

Future studies may be conducted to enhance the detection mechanism of the framework through identity-aware and graph-based approaches to future research. The enhanced detection mechanism would allow for greater identification of more complex links between vehicles and potential Sybil Attacks and would enhance the strength of the overall design.

Another aspect of strengthening the design will involve implementing complete post quantum cryptographic systems, using lattice based encryption methods, to assist researchers in evaluating the design's feasibility under real world conditions.

Moving forward, researchers may also look into expanding the federated learning components of the detected fraud system by incorporating more complicated models and architectures to include Deep Neural Networks (DNN), which would increase accuracy of the fraud detection system by allowing the system to adjust to changing patterns of fraud in real time.

Additionally, the effectiveness of the complete detection mechanism will need to be evaluated in large scale situations involving high density vehicular networks and the high frequency of handovers from one domain to another domain; however, the optimization of the overall framework using both energy aware and resource constrained optimization strategies would further improve its feasibility within the context of real world vehicular devices with lower computational resources.

## Data Availability

The raw data supporting the conclusions of this article will be made available by the authors, without undue reservation.
